# Separating the effects of water quality and urbanization on temperate insectivorous bats at the landscape scale

**DOI:** 10.1002/ece3.3693

**Published:** 2017-12-03

**Authors:** Han Li, Matina Kalcounis‐Rueppell

**Affiliations:** ^1^ Department of Biology The University of North Carolina at Greensboro Greensboro NC USA

**Keywords:** acoustic monitoring, bioindicators, insectivorous bat, landscape, urbanization, water quality

## Abstract

Many local scale studies have shown that bats respond to water quality degradation or urbanization in a species‐specific manner. However, few have separated the effects of urbanization versus water quality degradation on bats, in single city or single watershed case studies. Across North Carolina, USA, we used the standardized North American Bat Monitoring Program mobile transect protocol to survey bat activity in 2015 and 2016 at 41 sites. We collected statewide water quality and urban land cover data to disentangle the effects of urbanization and water quality degradation on bats at the landscape scale. We found that statewide, water quality degradation and urbanization were not correlated. We found that bats responded to water quality degradation and urbanization independently at the landscape scale. *Eptesicus fuscus* and *Lasiurus cinereus* negatively responded to water quality degradation. *Lasiurus borealis* and *Perimyotis subflavus* positively responded to water quality degradation. *Lasionycteris noctivagans* did not respond to water quality degradation but was more active in more urbanized areas. *Tadarida brasiliensis* positively responded to urbanization and was less active in areas with degraded water quality. We show that bat–water quality relationships found at the local scale are evident at a landscape scale. We confirm that bats are useful bioindicators for both urbanization and water quality degradation. We suggest that water quality can be used to predict the presence of bat species of conservation concern, such as *P. subflavus*, in areas where it has not been studied locally.

## 1 INTRODUCTION

Freshwater ecosystems are important to bats (Salvarina, 2016). Their importance to bats has been found not only in regions with an arid climate (e.g., Korine, Adams, Shamir, & Gross, 2015; Korine & Pinshow, 2004; Razgour, Korine, & Saltz, 2010; Williams & Dickman, 2004) but also in humid climates (Seibold, Buchner, Bässler, & Müller, 2013). Bats depend on open water as a source of drinking water. Freshwater ecosystems also serve as a food source because aquatic emergent insects are common prey for bats (Akasaka, Nakano, & Nakamura, 2009; Racey, Swift, Rydell, & Brodie, 1998). Additionally, foraging over water surfaces can be energetically beneficial for bats due to reduced commuting distance between food and drinking water sources (Kunz & Fenton, 2006; Mackey & Barclay, 1989; Siemers, Stilz, & Schnitzler, 2001). Therefore, the association between high bat activity and freshwater habitats has been documented worldwide at both local and landscape scales (e.g., Korine & Pinshow, 2004; Li & Wilkins, 2014; Razgour et al., 2010).

Since the industrial revolution, human activities have significantly altered the physical structure of water bodies, the inputs into water bodies, and the composition of natural biological communities in water bodies, leading to water quality degradation (Rai, Gaur, & Kumar, 1981; Schwarzenbach, Egli, Hofstetter, von Gunten, & Wehrli, 2010; Smith, Tilman, & Nekola, 1999). Water quality degradation can impact insectivorous bats in multiple ways. First, low‐quality water can contain a high concentration of toxins and cause toxin bioaccumulation through drinking water or consuming aquatic prey (Clarke‐Wood, Jenkins, Law, & Blakey, 2016; Jones, Jacobs, Kunz, Willig, & Racey, 2009; Naidoo, Vosloo, & Schoeman, 2013; Straka, Lentini, Lumsden, Wintle, & van der Ree, 2016; Zukal, Pikula, & Bandouchova, 2015). Second, water quality degradation caused by eutrophication or hypersaline pollutants can change the availability of drinkable water by changing water surface areas or water saline percentages (Cooper, 1993; Griffiths, Donato, Lumsden, & Coulson, 2014; Smith et al., 1999). Third, water quality can impact aquatic insect composition (either increase or decrease abundance of certain insects) and thus affect food source availability (Abbott, Sleeman, & Harrison, 2009; Akasaka et al., 2009; Kalcounis‐Rueppell, Payne, Huff, & Boyko, 2007; Park & Cristinacce, 2006).

Worldwide, many local scale studies have demonstrated species‐specific bat activity responses to water quality change (e.g., Clarke‐Wood et al., 2016; Kalcounis‐Rueppell et al., 2007; Korine et al., 2015; Naidoo et al., 2013; Vaughan, Jones, & Harris, 1996). Acoustic recordings have shown that there are species more active over less polluted water, such as *Eptesicus fuscus* (Kalcounis‐Rueppell et al., 2007), *Myotis capaccinii* (Biscardi et al., 2007), *M. daubentonii* (Abbott et al., 2009), and *M. dasycneme* (Sijpe et al., 2004). In contrast, other species have been found to be more active over polluted water, such as *Neoromicia nana* (Naidoo et al., 2013) and *Perimyotis subflavus* (Kalcounis‐Rueppell et al., 2007).

Water quality degradation can be caused by both point source, and nonpoint source, pollution (Smith et al., 1999). In many of the studies mentioned above, point sources in human settlements, such as sewage effluent or wastewater treatment plant effluent, were studied to quantify pollution or form a polluted versus nonpolluted pair experimental design (e.g., Kalcounis‐Rueppell et al., 2007; Naidoo et al., 2013; Vaughan et al., 1996). In these studies, water quality degradation was concomitant with urbanization gradients (Clarke‐Wood et al., 2016; Kalcounis‐Rueppell et al., 2007) making it difficult to disentangle the effects of water quality and urbanization. For example, in Kalcounis‐Rueppell et al. (2007), *E. fuscus* was found more active upstream of a wastewater treatment plant with high water quality. However, the study area in Kalcounis‐Rueppell et al. (2007) overlays a medium‐sized city with the upstream area being closer to the urbanized city center than the downstream area.

In addition to water quality, bats respond to urbanization in a species‐specific manner (Russo & Ancillotto, 2015). Urban habitats can provide roosts (e.g., Lausen & Barclay, 2006; Li & Wilkins, 2015; Neubaum, Wilson, & O'shea, 2007), food sources (e.g., Rydell, 1992; Williams, Mcdonnell, Phelan, Keim, & Van Der Ree, 2006), and drinking water sources (e.g., Bowles, Heideman, & Erickson, 1990; Razgour et al., 2010; Russo, Cistrone, & Jones, 2012). Many studies have shown that *E. fuscus* prefers urban downtown areas where it uses urban roosts (Duchamp, Sparks, & Whitaker, 2004; Li & Wilkins, 2014; Neubaum et al., 2007; Williams & Brittingham, 1997). In contrast, there are many bat species that avoid urban downtown areas due to the lack of vegetation or human bat conflicts (e.g., Duchamp et al., 2004; Threlfall, Law, & Banks, 2012, 2013a, 2013b). Furthermore, within urban areas, different bat species can show activity and distribution differences in response to urban spatial heterogeneity (Li & Wilkins, 2014; Luck, Smallbone, Threlfall, & Law, 2013).

The majority of bat activity–water quality relationship studies have occurred in a single city at the local scale without spatial replicates, and the local scale patterns might not scale up to a consistent pattern at the landscape scale. For example, local scale studies showed different responses of *M. daubentonii* to water quality degradation (negative, Abbott et al., 2009; neutral, Sijpe et al., 2004; and positive, Vaughan et al., 1996). Langton, Briggs, and Haysom (2010) demonstrated in a landscape modeling analysis that on average, *M. daubentonii* was negatively influenced by water quality degradation but site‐specific factors were also important. Furthermore, urbanization is not the only cause of water degradation. Nonpoint source pollution, such as agricultural runoff, could cause water quality degradation (Smith et al., 1999). Thus, there is a need to investigate whether activity differences in bats that relate to water quality degradation are because the bats are responding to water quality, the urban environment, or both.

Our objective was to disentangle the effects of water quality and urbanization on bat activity through a landscape‐scale analysis. Specifically, we wanted to determine whether patterns of relationships between water quality and species‐specific bat activity at a single stream scale would be evident at a landscape scale, independent of urbanization. We examined the effects of both urbanization and water quality on the common species that were previously examined in a single stream system and city, North Buffalo Creek in Greensboro, NC (Kalcounis‐Rueppell et al., 2007). We hypothesized that species‐specific bat activity would respond to water quality and urbanization independently at a landscape scale. Based on Kalcounis‐Rueppell et al. (2007), we predicted that at a landscape scale, *P. subflavus* would respond positively to water quality degradation. In contrast, *E. fuscus* would respond negatively to water quality degradation. Other species such as *Nycticeius humeralis* would not respond to water quality degradation. Based on previous literature (Li & Wilkins, 2014; Neubaum et al., 2007), we predicted that *E. fuscus* and *Tadarida brasiliensis* would respond positively to urbanization whereas other species, such as *N. humeralis* and *P. subflavus* would have no response.

## 2 METHODS

### 2.1 Sample site selection and transect mapping

The study area was the state of North Carolina, USA. We used the standardized bat sampling protocols from the North American Bat Monitoring Program (NABat, Loeb et al., 2015) to record bat activity. NABat divided the continental United States into 133,307 10 km by 10 km (100 km^2^) grid cells using a generalized random‐tessellation stratified (GRTS) master survey design algorithm (Loeb et al., 2015; Stevens & Olsen, 2004). The GRTS algorithm assigned a ranking number to each grid cell. The ranking system allowed subsampling of grid cells to be spatially balanced yet randomized (Larsen, Olsen, & Stevens, 2008; Stevens & Olsen, 2004). In this study, we used 100 top‐ranked GRTS grid cells in North Carolina as the candidate grid cells. We followed the GRTS ranking to choose cells as sample sites and excluded cells that met one or more of the following criteria: (1) the majority of the cell was in a neighbor state; (2) the cell did not have enough roads (e.g., cells overlaid by lakes or mountains); (3) the cell had limited night accessibility (such as military bases, parks that closed at dusk, or privately owned land).

In selected grid cells, we followed the protocols presented in Loeb et al. (2015) to map out an acoustic mobile transect survey (henceforth referred to as a “driving transect”) within each grid cell. The driving transect was a 30–35 km transect driven at 32 km/hr with low traffic volume and minimal stops (Loeb et al., 2015). We avoided gravel and dirt roads that were noisy, roads with low‐hanging vegetation, or roads that were extremely curvy. The driving transect passed through all common habitats within the grid cell. Driving transects were the same between 2015 and 2016 in the same cell. In 2016, major road construction caused two driving transects to be inaccessible. We considered these two grid cells not available and followed the GRTS ranking to replace them with the next two available ranked cells. Additionally, we could sample more grid cells following the GRTS ranking in 2016 because of additional resources. Each driving transect is considered as a sample site in this study.

### 2.2 Acoustic mobile transect survey

We conducted field work in June and July of 2015 and 2016. We used Anabat SD2 bat detectors (Titley Scientific, Australia) for driving transects. The detector was mounted on top of our vehicle using the Anabat Car Mount (Titley Scientific). The microphone was perpendicular to the road, facing straight up to the sky. The detector sensitivity was set between level 4 and level 5, which is a level that is suitable for species in the study area. All detectors involved in the project were calibrated with Anabat Equalizer (Titley Scientific), once each year, before each field season. The audio division ratio on the Anabat SD2 was set at 16. The data division ratio was set at 8.

Driving transects began 45 min after sunset and were only conducted on nights with no rain or fog and low wind speed (less than 10 km/hr). The driving transect route was mapped by a Mouse GPS unit (Titley Scientific). Each driving transect was sampled twice during each field season. The time gap between these two samples was less than 7 days. We coordinated sampling dates between years so that if a particular transect was driven in early June 2015, it was also driven in early June 2016. For each transect driven, we also collected the following metadata (driving transect covariates) in accordance with Loeb et al. (2015): total time of survey, temperature, relative humidity, wind speed, cloud cover, and moon phase.

**Figure 1:**
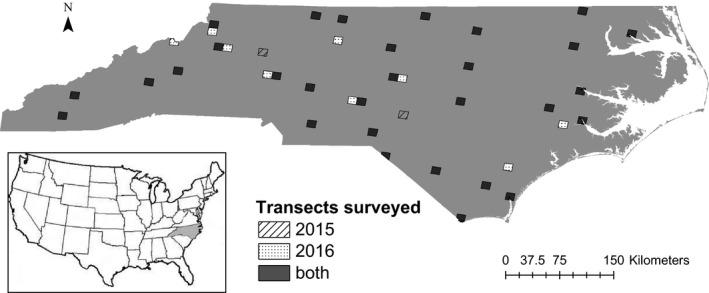
Study area map showing all North American Bat Monitoring Program (NABat) grid cells surveyed following a generalized random‐tessellation stratified master survey design in 2015 (strip), 2016 (dot), and both (solid) in the state of North Carolina, USA

We completed driving transect surveys in 32 NABat grid cells (32 sample sites, 64 nights of sampling) in 2015 and 39 grid cells (39 sample sites, 78 nights of sampling) in 2016. Thirty sample sites were sampled in both years. Two sites were only sampled in 2015 and nine sites were only sampled in 2016 (Figure 1). In total, we surveyed 41 sites across the state of North Carolina.

### 2.3 Bat acoustic species identification

All acoustic files recorded via Anabat SD2 detector were stored on compact flash (CF) cards. We used CFRead (Chris Corben, www.hoarybat.com) to download acoustic files and Analook (Chris Corben, www.hoarybat.com) to view the files. All acoustic files were first screened for bat pulse quality. Only files with at least three complete and clear pulses were selected for identification. Bat identification was conducted by comparing each pulse's characteristics (high frequency, low frequency, characteristic frequency, slopes, duration, and pulse gap) with a known bat call library (Kunz & Parsons, 2006; O'Farrell et al., 1999). The bat call library included calls collected by the authors (e.g., Li & Wilkins, 2014), calls presented in related acoustic publications (e.g., Buchler, 1980; Kurta et al., 2007; O'Farrell & Gannon, 1999), and reference bat calls from various bat acoustic analysis workshops.

We used a conservative approach to conduct bat species identification. First, all identifications were conducted manually by the first author for consistency. The species considered are listed as follow (species abbreviation used in all tables and figures): big brown bat (*Eptesicus fuscus*, EPFU), eastern red bat (*Lasiurus borealis*, LABO), hoary bat (*Lasiurus cinereus*, LACI), silver‐haired bat (*Lasionycteris noctivagans*, LANO), evening bat (*Nycticeius humeralis*, NYHU), tricolored bat (*Perimyotis subflavus*, PESU), and Mexican free‐tailed bat (*Tadarida brasiliensis*, TABR). Secondly, we only identified a call file to species when the unique characteristics (high frequency, low frequency, characteristic frequency, slopes, duration, and pulse gap) of the species were found in multiple pulses. Certain species, such as *L. borealis* and *N. humeralis*, might generate pulses that are hard to differentiate. If a call file only included pulses that were hard to differentiate, we did not identify the call file to species. Thirdly, we only identified species with statewide ranges and we did not summarize all species together as total bat activity for any analysis, even though this variable was considered in the previous local scale study (Kalcounis‐Rueppell et al., 2007). We made this decision because there are certain species in North Carolina that do not have a statewide distribution, and therefore, total number of bat calls of all species would be biased in certain regions. Lastly, we only conducted species‐specific analysis and did not compare among species as acoustic recordings and identification might be biased toward certain species (Russo & Voigt, 2016).

As all driving transects were similar in distance, speed, and length of driving time, we summarized bat activity as the number of bat calls per transect for each species. The two transect nights sampled for each grid cell, in each year, were averaged to reduce temporal autocorrelation (Wright, Irvine, & Rodhouse, 2016).

### 2.4 Urban land cover data

We characterized urban development using the National Land Cover Database 2011 (NLCD 2011, Homer et al., 2015) and calculated the percentage of land categorized as “urban development” at each sample site. We used ArcMap (10.4.1, ESRI, California) to generate a 5‐km‐radius buffer along each of our 41 sample sites (around each driving transect). We selected 5 km as the buffer radius because it represents the active range of bat species involved in this study (Barclay, 1985; Kunz & Fenton, 2006; Norberg, 1990).

To calculate the percentage of land, we used buffers to extract land cover raster images from NLCD 2011 and generated Tag Image File Format (TIFF) files in ArcMap. We then used FRAGSTATS (McGarigal, Cushman, & Ene, 2012) to extract the land cover percentages from TIFF files. Within NLCD 2011, there are four categories of urban development: open space (e.g., isolated houses), low intensity (e.g., single‐family house residential communities), medium intensity (e.g., low‐rise apartment buildings, shopping areas), and high intensity (e.g., high‐rise office or apartment buildings). Correlation analysis showed that within the buffers, the percentages of each land cover type were correlated (all pairs variance inflation factors >3; Zuur, Ieno, Walker, Saveliev, & Smith, 2009). Thus, we summed percentages from each category into one variable called “urban land cover” for each of our 41 sample sites.

### 2.5 Water quality data

The statewide water quality data were provided by the North Carolina Department of Environmental Quality, Division of Water Resources (DWR), Biological Assessment Branch. DWR routinely (2–3 years as a sampling cycle) samples freshwater benthic macroinvertebrate communities and evaluates biological integrity. The protocols rate water quality in bioclassification ratings based on macroinvertebrate diversity and abundance, along with water chemistry analyses, ambient toxicity data, and habitat evaluations (Chapman, 1996; North Carolina Department of Environmental Quality Division of Water Resources Biological Assessment Branch, 2015). The five ratings are as follows: “excellent”, “good”, “good‐fair”, “fair”, and “poor”, an order reflecting decreasing water quality based on the benthic community and other environmental variables.

**Figure 2:**
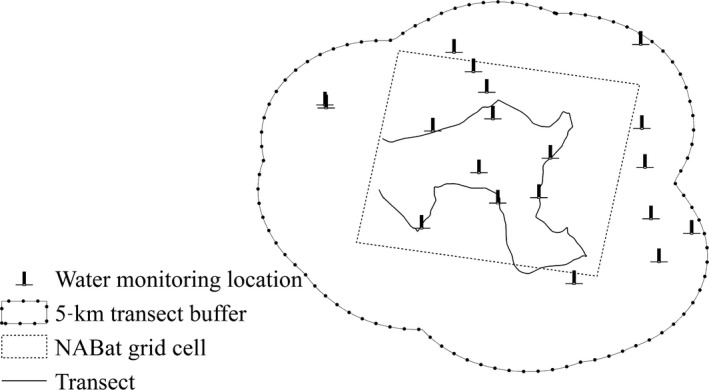
An example of the spatial relationship between the North American Bat Monitoring Program (NABat) grid cell (dash line), the mobile transect driven to sample bats (solid line), the 5‐km‐radius buffer (solid line with dot) along the transect for urban land cover, and water monitoring locations used for water quality (beacon)

At each sample site, we used the 5‐km‐radius buffer along each transect to include all water sampling locations. In total, 593 water sampling locations were included among 41 sample sites (Figure 2). The minimum number of water sampling locations per buffer was 5 and the maximum was 40. We only used the most current water quality rating (from 2014 to 2016) for each water sampling location. As each buffer had multiple water sampling locations, we selected the mode of all water quality ratings as the indicator of water quality.

### 2.6 Statistical analysis

Before testing the hypothesis on the effects of water quality and urban land cover, we conducted preliminary analyses to examine (1) the effects of driving transect covariates on bat activity; and (2) differences in species‐specific activity between 2015 and 2016. We constructed species‐specific generalized linear models (GLM) for each covariate.

Our overall goal was to determine the effects of independent variables instead of constructing predictive models. The bat activity data showed high variance–mean ratios (all larger than 2) suggesting large variance and data overdispersion. Therefore, we chose a Bayesian approach and conducted Markov Chain Monte Carlo (MCMC) stochastic simulation sampling for more accurate inferences. In this way, we examined many GLMs based on simulations to see whether there was a constraining pattern in the data that caused models to converge to the same posterior distribution (Barr, Levy, Scheepers, & Tily, 2013; Martin, Quinn, & Park, 2011; McCarthy, 2007). In this modeling approach, a posterior distribution of the GLM regression estimate was generated. Instead of evaluating one *p* value for one regression estimate, the posterior mean of simulated regression estimates and its 95% confidence interval (CI) were examined. If the posterior mean's 95% CI did not overlay with 0, data converged and there was a significant relationship (Martin et al., 2011). Positive posterior means indicate positive relationships and negative posterior means indicate negative relationships.

When constructing the GLM, we modeled the data with a negative binomial distribution due to data overdispersion (Frühwirth‐Schnatter, Frühwirth, Held, & Rue, 2009; Martin et al., 2011). For the prior in Bayesian modeling, we constructed models with priors suggested in literature suitable for large posterior variance (Hadfield, 2015; McCarthy, 2007; Yang & Berger, 1996) and completed 20,000 runs of simulations and extracted 2,000 simulations to evaluate posterior distributions (thinning interval 10). The modeling was completed in R (version 3.4.1, R Development Core Team, 2008) using package MCMCpack (Martin et al., 2011).

We only report the results of significant covariates. Nonsignificant covariates were not included in further analysis and not reported. In another preliminary analysis, we checked for collinearity between water quality and urban land cover with a multinomial regression model. Based on the regression model, we used Wald's test to calculate *p* values of pairwise comparisons between the water quality category “excellent” and all other categories. Any *p* value <0.05 would indicate that water quality responded to urban land cover. This analysis was completed in R using package nnet (Venables & Ripley, 2002).

To test the hypotheses about the effects of water quality and urban land cover on bat activity, we used the MCMC simulation modeling technique described above. We constructed species‐specific GLMs with bat activity as the dependent variable and water quality or urban land cover as the independent variable. Due to the limited sample size, we constructed models separately for water quality and urban land cover to avoid unstable models (Quinn & Keough, 2002; Sheather, 2009; see supporting information for model stability graphs) and we did not investigate the interaction. As year had an effect on *N. humeralis* activity, we included year in the models of this species. For all other species, we pooled data from both years for the GLM.

## 3 RESULTS

We collected 9,716 files that included bat echolocation pulses. There were 5,233 files that met our identification criteria and we could identify 3,978 call files to species. *L. borealis* was the most common species (1,508 files), followed by *N. humeralis* (856 files), *P. subflavus* (552 files), *E. fuscus* (420 files), *L. noctivagans* (310 files), *T. brasiliensis* (228 files), and *L. cinereus* (104 files).

**Figure 3:**
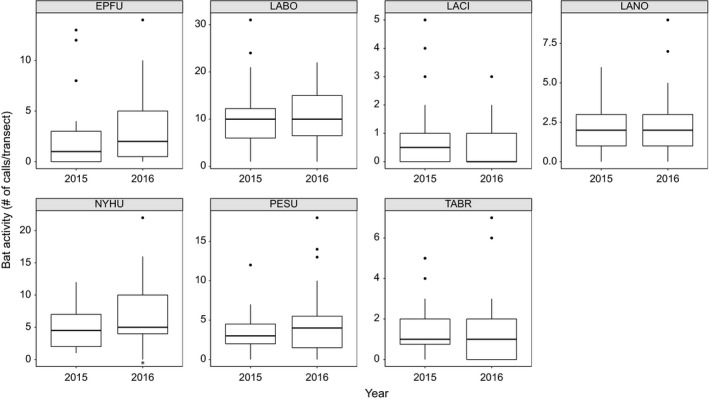
Species‐specific relationships between bat activity and year. The species abbreviations are as follows: *Eptesicus fuscus*, EPFU; *Lasiurus borealis*, LABO; *Lasiurus cinereus*, LACI; *Lasionycteris noctivagans*, LANO; *Nycticeius humeralis*, NYHU; *Perimyotis subflavus*, PESU; and *Tadarida brasiliensis*, TABR. Significant difference is indicated by *. More NYHU calls were recorded in 2016

We found no relationship between species‐specific bat activity and the following survey covariates: total time of survey, temperature, relative humidity, wind speed, cloud cover, and moon phase. However, there was significantly higher *N. humeralis* activity in 2016 than in 2015 (Table 1; Figure 3). The GLM MCMC simulation generated a positive regression estimate posterior mean 0.436 (*n* = 71).

**Table 1:** Generalized linear model (GLM) results using Markov Chain Monte Carlo simulation modeling bat activity against year, *n* = 71

Species	Posterior mean	Lower 95% CI	Upper 95% CI
EPFU	0.212	−0.394	0.819
LABO	0.089	−0.178	0.359
LACI	−0.563	−1.208	0.077
LANO	0.215	−0.201	0.660
NYHU*	0.436	0.116	0.744
PESU	0.205	−0.159	0.594
TABR	−0.017	−0.544	0.494

The year 2016 was compared to 2015. If the regression estimate's 95% confidence interval (CI) overlays with 0, the relationship in GLM is not significant. Significant posterior means are noted by * in the species column.

**Figure 4:**
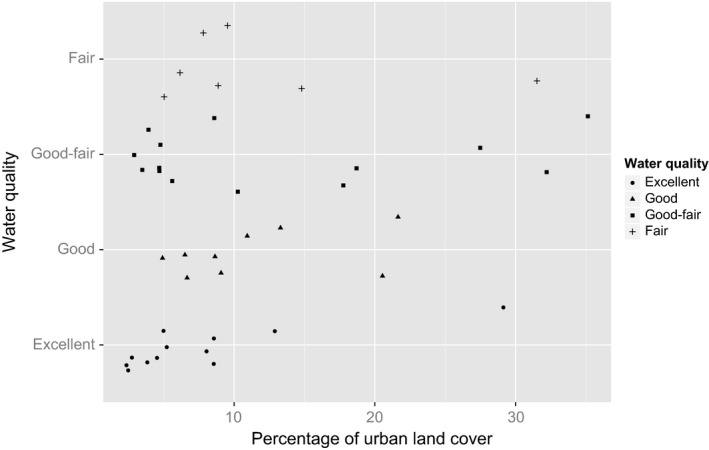
The relationship between water quality and urban land cover for 41 North American Bat Monitoring Program grid cells sampled in the study. There was no change in water quality category (excellent: circle, good: triangle, good‐fair: square, fair: cross) as the percentage urban land cover increased in the landscape, based on multinomial regression

Among 41 sample sites, urban land cover ranged between 2.4% and 35.1% with a mean and standard error of 10.5 ± 8.6%. Among these sites, 11 had a water quality mode of “excellent,” nine of “good,” 14 of “good‐fair,” and seven of “fair”. No sample site had a water quality mode of “poor”. The multinomial regression model Wald's tests showed no correlation between water quality and percentage of urban development land cover (Table 2; Figure 4).

**Table 2:** Multinomial regression results modeling water quality change against urban land cover, *n* = 41

Water quality	Coefficient	*SE*	*z*	*p *> |*z*|
Good ~ excellent	0.066	0.063	1.044	0.296
Good‐fair ~ excellent	0.083	0.058	1.421	0.155
Fair ~ excellent	0.073	0.065	1.119	0.262

Comparisons were made between “excellent” and other water quality categories.

### 3.1 The effect of water quality on bat activity

**Figure 5:**
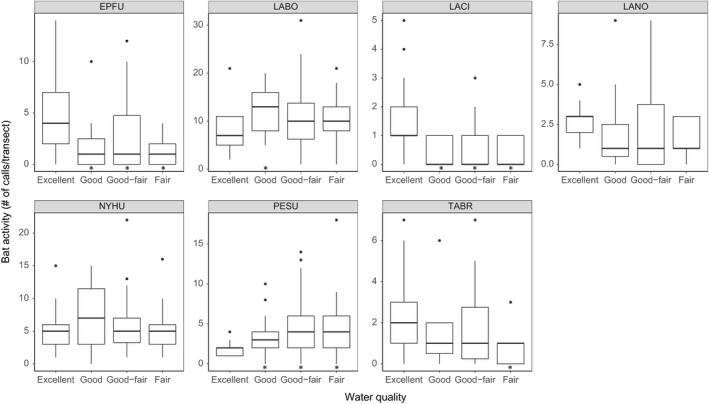
Species‐specific relationships between bat activity and water quality. The species abbreviations are as follows: *Eptesicus fuscus*, EPFU; *Lasiurus borealis*, LABO; *Lasiurus cinereus*, LACI; *Lasionycteris noctivagans*, LANO; *Nycticeius humeralis*, NYHU; *Perimyotis subflavus*, PESU; and *Tadarida brasiliensis*, TABR. Generalized linear models compared bat activity at sites with good, good‐fair, or fair water quality with bat activity at sites with excellent water quality. Significant difference is indicated by *. EPFU and LACI activity was lower at good, good‐fair, or fair sites as compared to excellent sites. TABR activity was lower at fair sites as compared to excellent sites. PESU activity was higher at good, good‐fair, or fair sites as compared to excellent sites. LABO activity was higher at good sites as compared to excellent sites

The effect of water quality on bat activity is varied by species (Table 3; Figure 5). Three bat species showed a significant negative response to water quality degradation. *E. fuscus* activity was approximately three times, 1.9 times, and 3.6 times higher when comparing water quality category “excellent” to “good,” “good‐fair,” and “fair,” respectively. *L. cinereus* activity was approximately five times, 4.3 times, and 4.2 times higher when comparing water quality category “excellent” to “good,” “good‐fair,” and “fair,” respectively. *T. brasiliensis* activity was approximately 2.6 times higher at “excellent” water sites than at “fair” water sites.

**Table 3:** Generalized linear model (GLM) results using Markov Chain Monte Carlo simulation modeling bat activity against water quality, *n* = 71

Species	Water quality	Posterior mean	Lower 95% CI	Upper 95% CI
EPFU	Good ~ excellent*	−1.106	−1.899	−0.295
	Good‐fair ~ excellent*	−0.637	−1.333	−0.058
	Fair ~ excellent*	−1.301	−2.208	−0.410
LABO	Good ~ excellent*	0.420	0.035	0.796
	Good‐fair ~ excellent	0.349	−0.010	0.714
	Fair ~ excellent	0.310	−0.122	0.727
LACI	Good ~ excellent*	−1.632	−2.724	−0.694
	Good‐fair ~ excellent*	−1.146	−1.866	−0.441
	Fair ~ excellent*	−1.553	−2.607	−0.586
LANO	Good ~ excellent	−0.338	−0.949	0.291
	Good‐fair ~ excellent	−0.222	−0.698	0.269
	Fair ~ excellent	−0.595	−1.295	0.117
NYHU	Good ~ excellent	0.293	−0.190	0.786
	Good‐fair ~ excellent	0.134	−0.261	0.539
	Fair ~ excellent	0.105	−0.383	0.602
PESU	Good ~ excellent*	0.603	0.017	1.161
	Good‐fair ~ excellent*	0.868	0.369	1.380
	Fair ~ excellent*	0.901	0.296	1.500
TABR	Good ~ excellent	−0.468	−1.187	0.193
	Good‐fair ~ excellent	−0.217	−0.802	0.345
	Fair ~ excellent*	−0.951	−1.822	−0.089

Comparisons were made between “excellent” and other water quality categories. If the regression estimate's 95% confidence interval (CI) overlays with 0, the relationship in GLM is not significant. Significant posterior means are noted by * in the water quality column to indicate the significant pair.

In contrast, two species showed a significant positive response to water quality degradation (Table 3; Figure 5). *P. subflavus* activity was approximately 2.5 times, 2.3 times, and 1.8 times higher in sites with water quality categories of “fair,” “good‐fair,” and “good,” respectively, when compared to “excellent”. *L. borealis* activity was approximately 1.5 times higher at “good” water sites than at “excellent” water sites. We did not find any significant relationship between activity of *Lasionycteris noctivagans* or *N. humeralis* and water quality.

### 3.2 The effect of urbanization on bat activity

**Figure 6:**
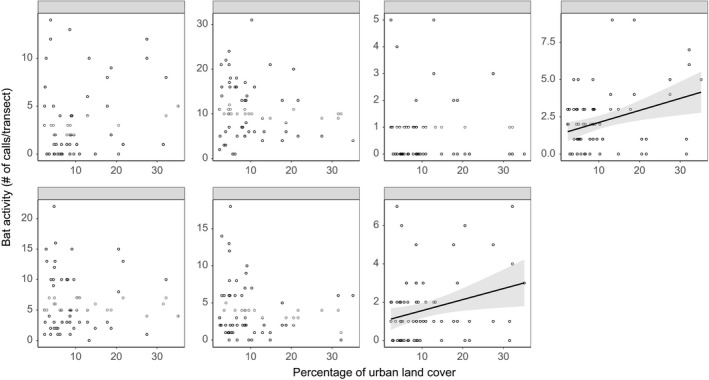
Species‐specific relationships between bat activity and urban land cover. Shaded areas represent 95% confidence intervals around the trend lines estimated by generalized linear models. Only significant relationship trend lines are plotted. The species abbreviations are as follows: *Eptesicus fuscus*, EPFU; *Lasiurus borealis*, LABO; *Lasiurus cinereus*, LACI; *Lasionycteris noctivagans*, LANO; *Nycticeius humeralis*, NYHU; *Perimyotis subflavus*, PESU; and *Tadarida brasiliensis*, TABR. Generalized linear models suggested that only LANO and TABR activity consistently increased as more urban land cover was present in the 5‐km‐radius buffer of a site

Of seven bat species we modeled, only two, *L. noctivagans* and *T. brasiliensis*, had a significant relationship with urban land cover (Table 4; Figure 6). Both species positively responded to urban land cover, indicating higher bat activity in sites higher proportions of urban land cover. For the other five species, our simulation models did not find converging outcomes. Therefore, we could not conclude that urban land cover had an effect on the activity of these species.

**Table 4:** Generalized linear model (GLM) results using Markov Chain Monte Carlo simulation modeling bat activity against urban land cover, *n* = 71

Species	Posterior mean	Lower 95% CI	Upper 95% CI
EPFU	0.024	−0.008	0.060
LABO	−0.010	−0.026	0.007
LACI	0.014	−0.025	0.054
LANO*	0.032	0.011	0.053
NYHU	−0.002	−0.019	0.017
PESU	−0.015	−0.037	0.009
TABR*	0.030	0.007	0.053

If the regression estimate's 95% confidence interval (CI) overlays with 0, the relationship in GLM is not significant. Significant posterior means are noted by * in the species column.

## 4 DISCUSSION

We were able to disentangle the effects of water quality and urbanization on bat activity and examine their impacts separately, through our landscape‐scale analysis. At the landscape scale, we did not find any correlation between water quality and urban land cover likely because anthropogenic pollution sources in nonurban areas also affect water quality (Brabec, Schulte, & Richards, 2002; Smith et al., 1999). For example, manure runoff from industrialized farms and mining waste in nonurban areas are both significant factors in water quality degradation (Cooper, 1993; Griffiths et al., 2014; Hooda, Edwards, Anderson, & Miller, 2000; Rai et al., 1981). Additionally, modern urban design and planning tend to place industrial zones, with heavy pollutants, in rural environments (Antrop, 2004; Jepson & Edwards, 2010; Ning & Yan, 1995). Even though municipal sewage effluent and runoff from impervious surfaces in cities can cause water quality degradation, at the landscape scale, water quality degradation and urbanization are two different anthropogenic processes and we discuss each below.

### 4.1 The effect of water quality on bat activity

At the landscape scale, we found that *E. fuscus* and *L. cinereus* were more active, whereas *P. subflavus* was less active, in areas with higher water quality and that *N. humeralis* did not respond to water quality degradation. There could be multiple mechanisms that explain the patterns that we found at the landscape scale that include prey availability, drinking water availability, and toxicity. Aquatic insect availability changes in response to water quality degradation, and there are demonstrated species‐specific responses to water quality degradation (e.g., Abbott et al., 2009; Kalcounis‐Rueppell et al., 2007; Wickramasinghe, Harris, Jones, & Vaughan, 2003; Wickramasinghe, Harris, Jones, & Vaughan Jennings, 2004). Other mechanisms through which water quality degradation can affect bats are toxins or other chemical bioaccumulation (e.g., Clarke‐Wood et al., 2016; Korine et al., 2015) and/or drinking water availability (e.g., Cooper, 1993; Griffiths et al., 2014). It is important to note that mechanisms we mention above can interact and co‐impact bats. For example, aquatic insects may attract bats to prey and increase bat activity temporarily. However, in the long term, toxic bioaccumulation can lead to population decreases (Naidoo et al., 2013; Zukal et al., 2015). Future studies should investigate the underlying mechanisms to explain patterns of bat activity and water quality at the landscape scale.

Our results at the landscape scale are consistent with the results found by Kalcounis‐Rueppell et al. (2007) at the local scale, as we hypothesized. Specifically, both studies found *E. fuscus* and *L. cinereus* to be more active in areas with higher water quality and *P. subflavus* to be more active in areas with water quality degradation. Kalcounis‐Rueppell et al. (2007) sampled insects and demonstrated prey availability to be a mechanism to explain species‐specific bat responses to water quality degradation and it is possible that the same mechanism can explain our landscape‐scale concordant results; however, this would require additional studies. Regardless of whether the mechanisms that explain the landscape and local patterns are the same, we show that local scale studies are relevant at the landscape scale. The bat activity–water quality patterns we identified at the landscape, that are concordant with Kalcounis‐Rueppell et al. (2007), reinforce bats as good bioindicators for water quality degradation. In particular, high *P. subflavus* activity would indicate low water quality.

It is important to consider the impact of water availability on our conclusions. Water availability was likely not a factor in species‐specific patterns of response to degradation because North Carolina has a humid subtropical climate (Robinson, 2015). More importantly, we analyzed whether water availability limits bat species distribution in North Carolina and found no evidence for this.

Water quality degradation can potentially cause local extirpation via directly limiting resources or creating ecological traps (e.g., Clarke‐Wood et al., 2016; Naidoo et al., 2013). Both our results and those of Kalcounis‐Rueppell et al. (2007) show that certain species of bats were negatively impacted by low‐quality water. The long‐term impacts of this pattern will require further studies to examine physiological and reproductive impacts on individuals of these species as in Naidoo et al. (2013). However, given the availability of water in North Carolina, it is possible that bats which were negatively impacted by low water quality could find alternative water sources elsewhere on the landscape. More long‐term population trend data and individual physiological/reproductive data would be needed to evaluate whether water quality degradation is a major, long‐term, threat to bats in our study area.

### 4.2 The effect of urbanization on bat activity

Urbanization has been reported to negatively impact bats (e.g., Russo & Ancillotto, 2015; Threlfall et al., 2012, 2013a, 2013b) and correlations between bat species activity and urban land cover at the landscape scale support bats as bioindicators of urbanization (Russo & Ancillotto, 2015). However, our study did not find any bat species that were negatively associated with urban land cover. Instead, we found that activity of *T. brasiliensis* and *L. noctivagans* was positively correlated to urban land cover, and contrary to our expectations, *E. fuscus* activity was not related to urban land cover. One explanation for not finding a negative association between bats and urbanization has to do with detectability of forest interior species from the genus *Myotis*. Our driving transect method does not sample forest interior species well. A second reason also has to do with limitations of our driving transect method because we were not able to sample major urban centers due to logistic constraints of the driving transect protocol. For example, one cannot drive through an urban center without stopping. Thus, long‐term monitoring of bats via different survey methods (e.g., walking transects, stationary transects in urban centers) is needed to improve our understanding of the effects of urbanization on all bats, especially species that can be negatively impacted by urbanization.

We found a positive relationship between *T. brasiliensis* and urban land cover, as expected. *T. brasiliensis* uses various man‐made structures as roosts in urban environments and has the potential to roost in large colonies (Davis, Herreid, & Short, 1962; Fraze & Wilkins, 1990; Li & Wilkins, 2015; Wilkins, 1989). The pattern we found is consistent with other studies of *T. brasiliensis* and urbanization. In addition, we found that *L. noctivagans* was positively correlated to urban land cover. Less is known about *L. noctivagans* and urbanization; however, *L. noctivagans* was positively correlated to urbanization in the Chicago area (Gehrt & Chelsvig, 2004, but see Dixon, 2011). As *L. noctivagans* is considered a tree‐roosting species (Cryan, 2003), it is unlikely that urban areas provide additional roost sites as with *T. brasiliensis*; however, there may be foraging resources (prey or habitat) that are enhanced in urban areas. Further studies are needed to better understand the urban ecology of *L. noctivagans*.

Although *E. fuscus* has been shown to prefer urban areas where it uses urban roosts (Duchamp et al., 2004; Neubaum et al., 2007; Williams & Brittingham, 1997), we did not find any relationship between *E. fuscus* and urban land cover. One reason for the discrepancy may be that in our study area, *E. fuscus* uses urban roosts but prefers to commute to outside of the city, or to inner‐city forested spaces (such as greenways or parks) to forage (Dixon, 2011; Duchamp et al., 2004; Lausen & Barclay, 2006; Neubaum et al., 2007). Alternatively, there may an effect of the presence of *T. brasiliensis* on the activity of *E. fuscus* in urban areas. In studies of urban bats in Texas and California, there is evidence that *E. fuscus* is not as prevalent as *T. brasiliensis* when both species coexist in urban environments (Krauel & LeBuhn, 2016; Li & Wilkins, 2014). The proposed mechanism is that *T. brasiliensis* may outcompete *E. fuscus* in urban areas. Interestingly, *T. brasiliensis* was one of two species that were positively correlated to urbanization in our study. Further studies should focus on a broader scale to compare these two species’ association with urban environments in sympatry.

In conclusion, our study is the first to disentangle the effects of urbanization and water degradation on bats at the landscape scale. We show that water quality degradation and urbanization can negatively or positively impact certain species at the landscape scale. Species‐specific responses to water quality degradation and urbanization need to be considered in conservation planning. We also demonstrate, for the first time, that the effects of water degradation on bats at a local scale are also evident at the landscape scale. The concordance between scales underscores the important contribution that local scale studies of water quality and urbanization make to understanding bat biology. Future studies should examine mechanisms that regulate how bat responses scale up from local to landscape scales. Results from this, and other studies, show that bats are useful bioindicators for both urbanization and water degradation. Interestingly, our work can also inform the local scale from the landscape scale. For example, we should be able to predict the presence probability of *P. subflavus* in areas where it has not been studied locally based on water quality information. This is relevant because *P. subflavus* is a species that is experiencing severe population decline caused by white‐nose syndrome and needs conservation actions (Frick et al., 2015; Langwig et al., 2012).

## CONFLICT OF INTEREST

Authors declared no conflict of interest involved.

## AUTHOR CONTRIBUTIONS

M. Kalcounis‐Ruppell's sole contributions included funding acquisition, conceiving the project, and working with the North Carolina Department of Environmental Quality, Division of Water Resources, Biological Assessment Branch for acquiring water quality data. H. Li's sole contributions included bat acoustic data collection and identification, land cover data analysis, and statistical analysis. For other aspects of the production of this manuscript, H. Li and M. Kalcounis‐Ruppell contributed equally.

## DATA ACCESSIBILITY

Raw data are deposited to the Dryad Digital Repository.
