# Supplementary material for: Separating the effects of water quality and urbanization on temperate insectivorous bats at the landscape scale
Source: Ecol Evol. 2017 Dec 3;8(1):667–78. doi: 10.1002/ece3.3693 (PMC5756845; doi:10.1002/ece3.3693)

Eptesicus fuscus

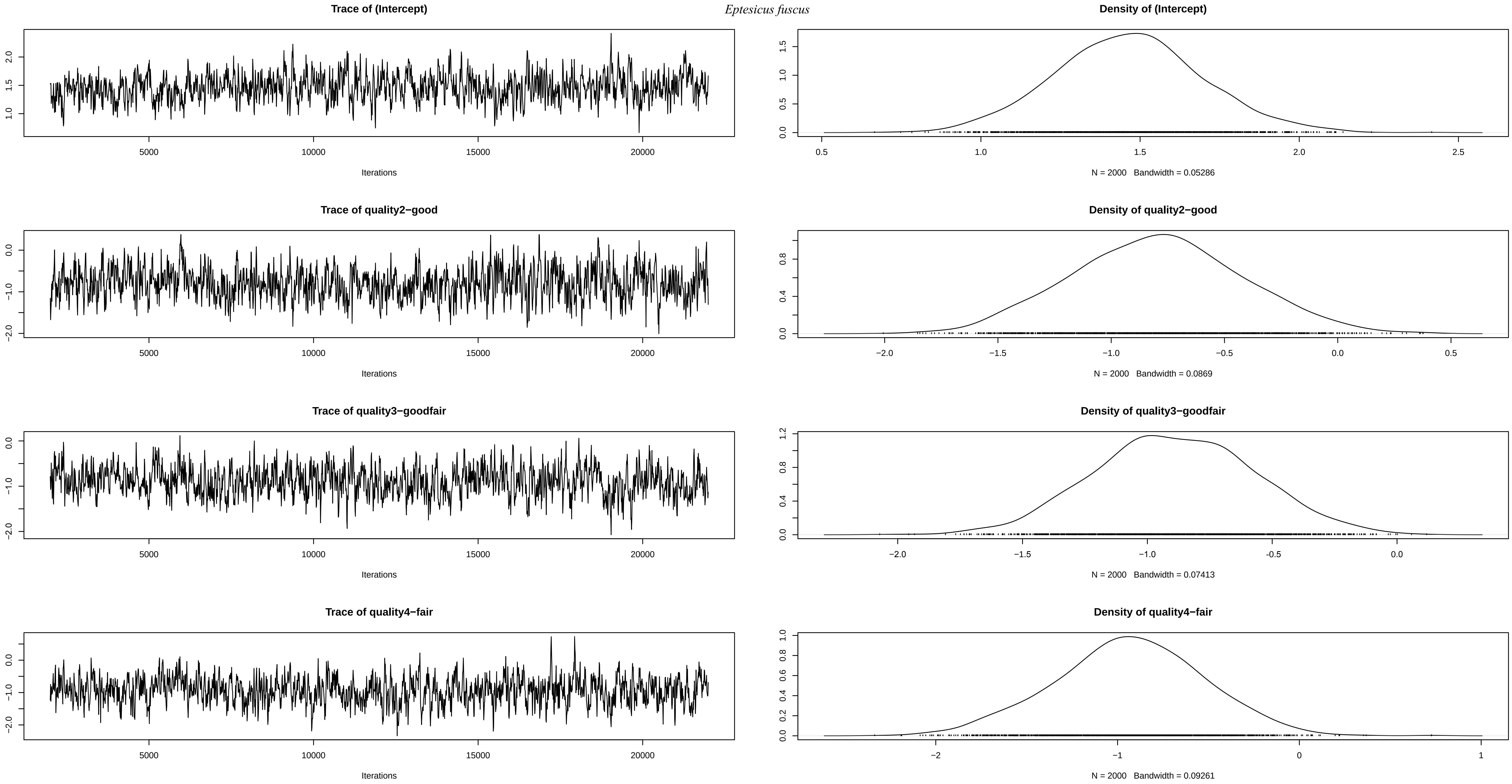

Lasiurus borealis

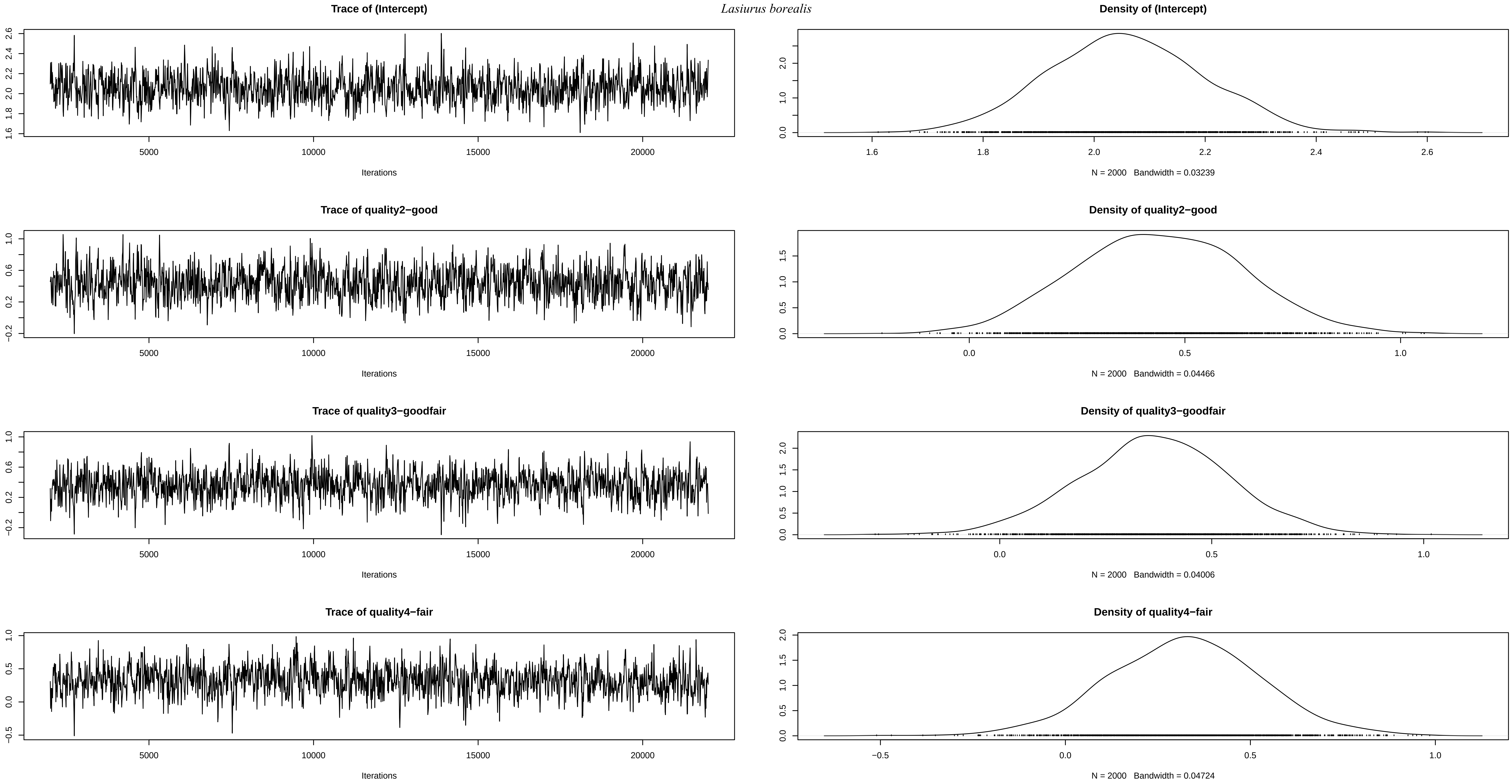

Lasiurus cinereus

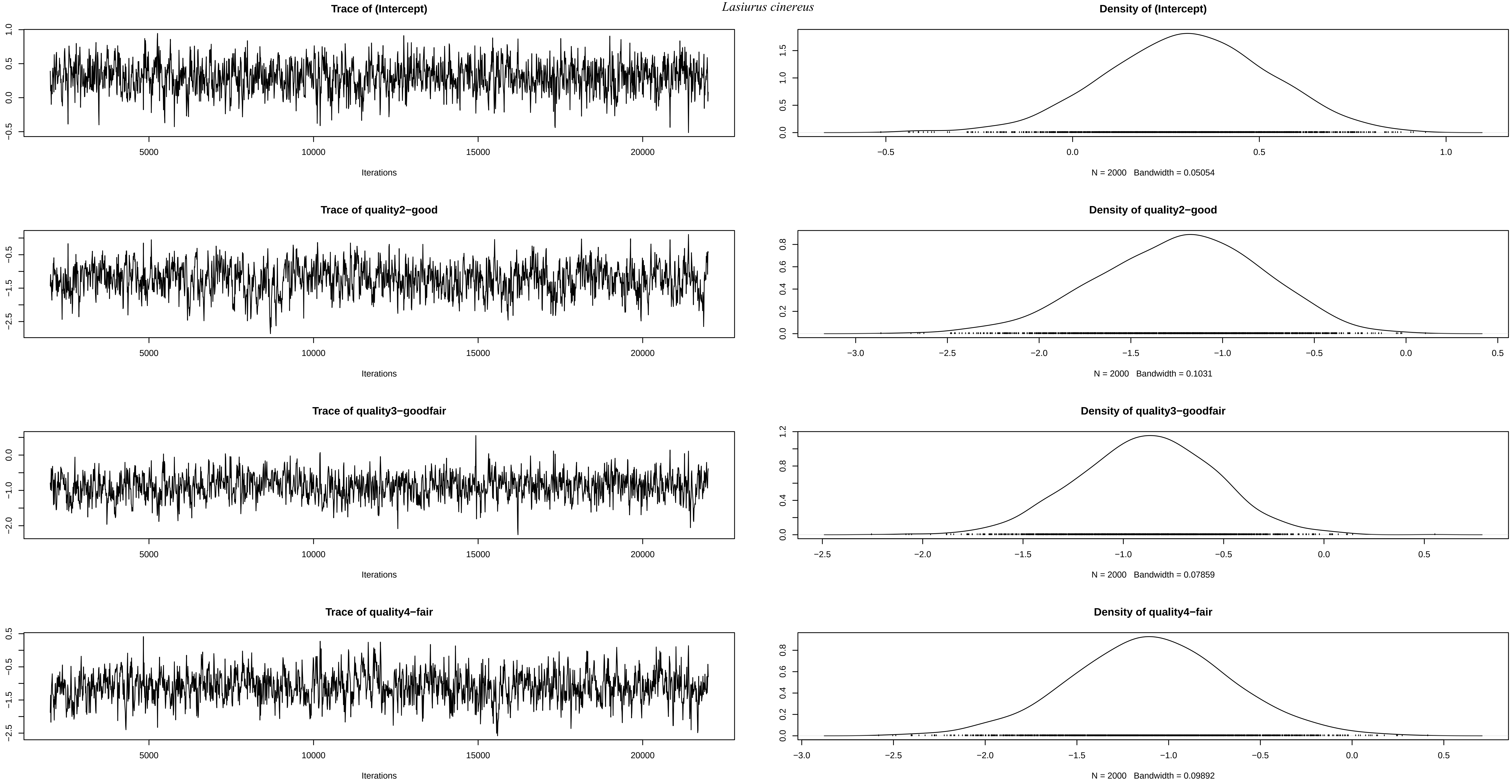

Lasionycteris noctivagans

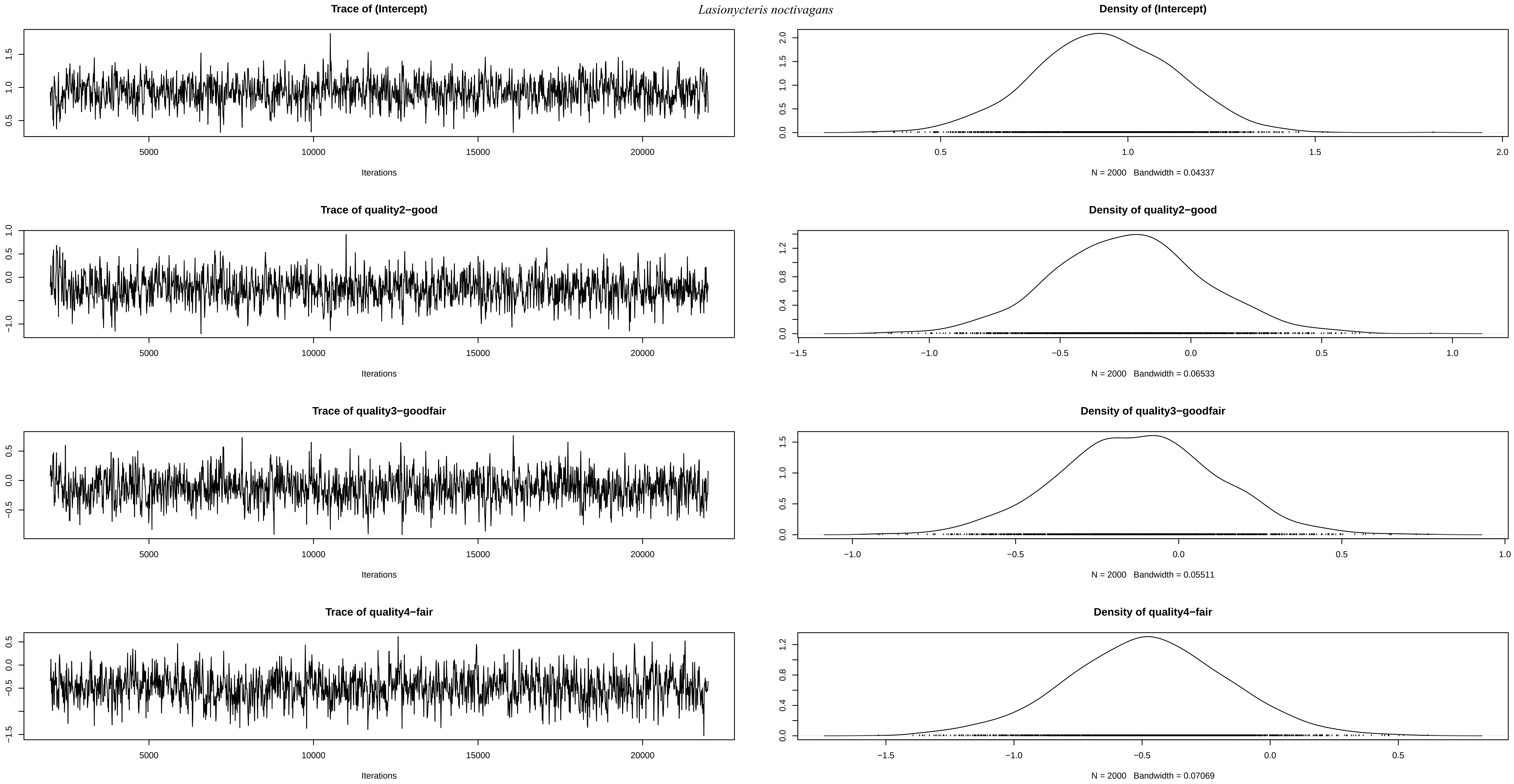

Nycticeius humeralis

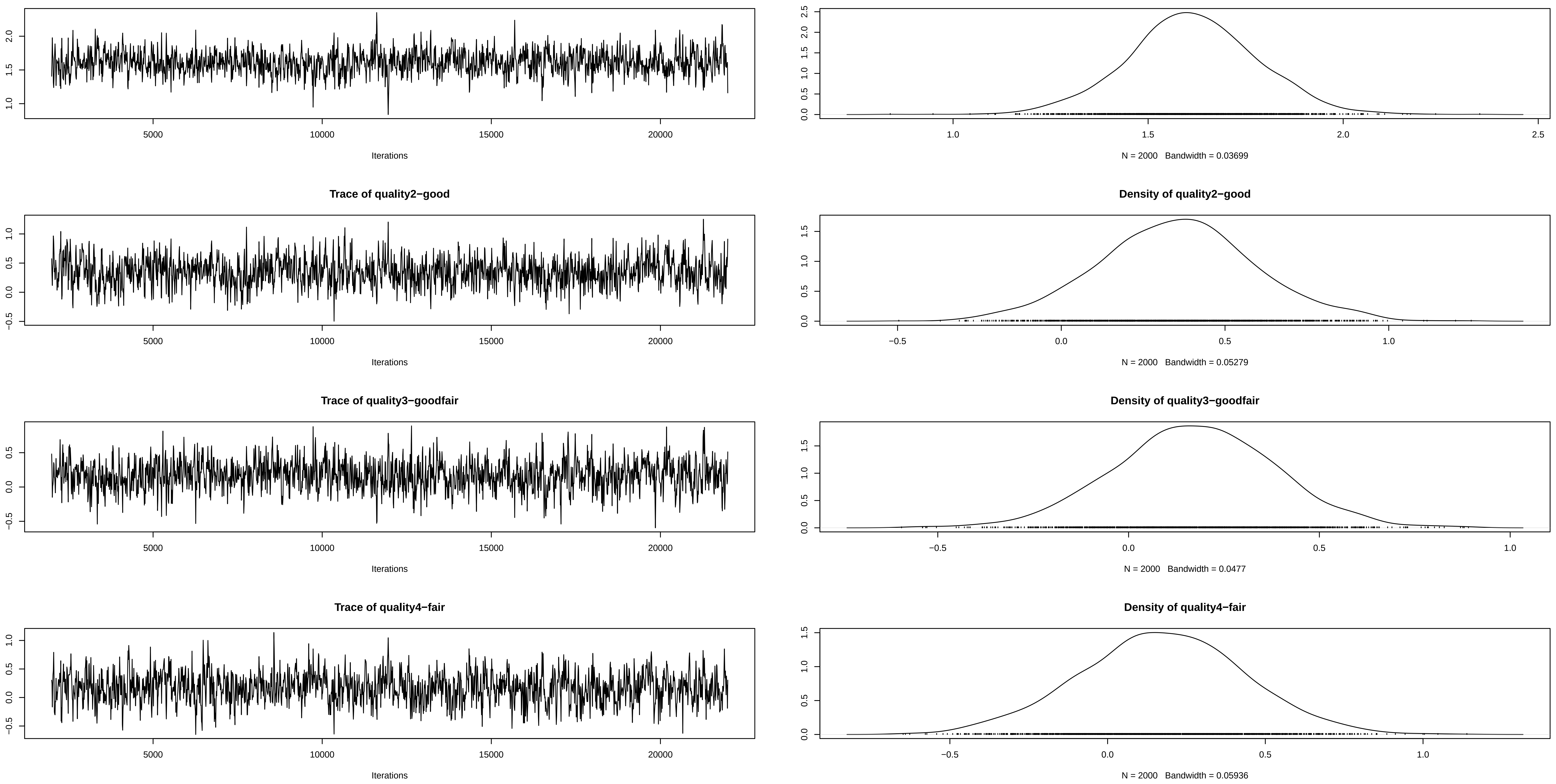

Perimyotis subflavus

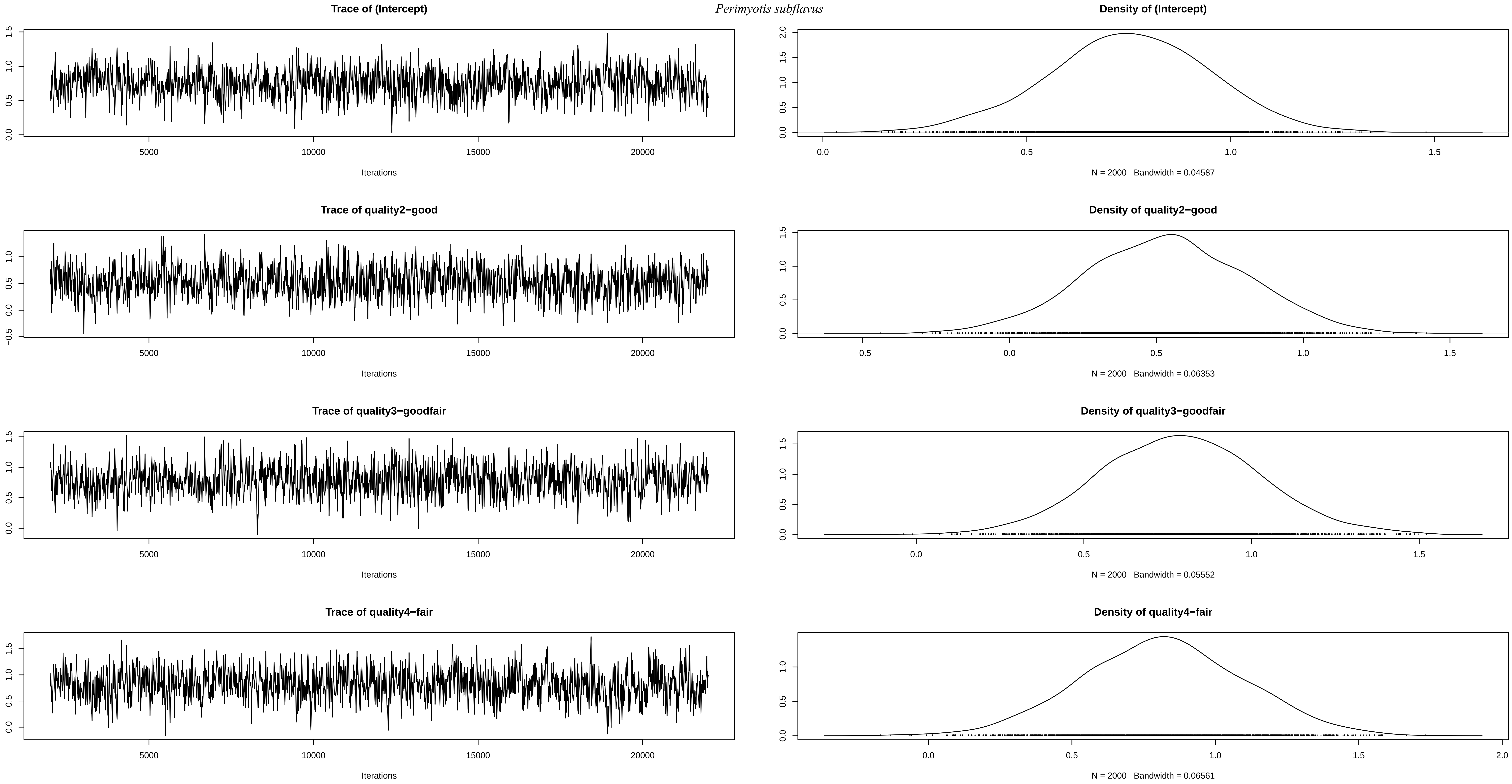

Tadarida brasiliensis

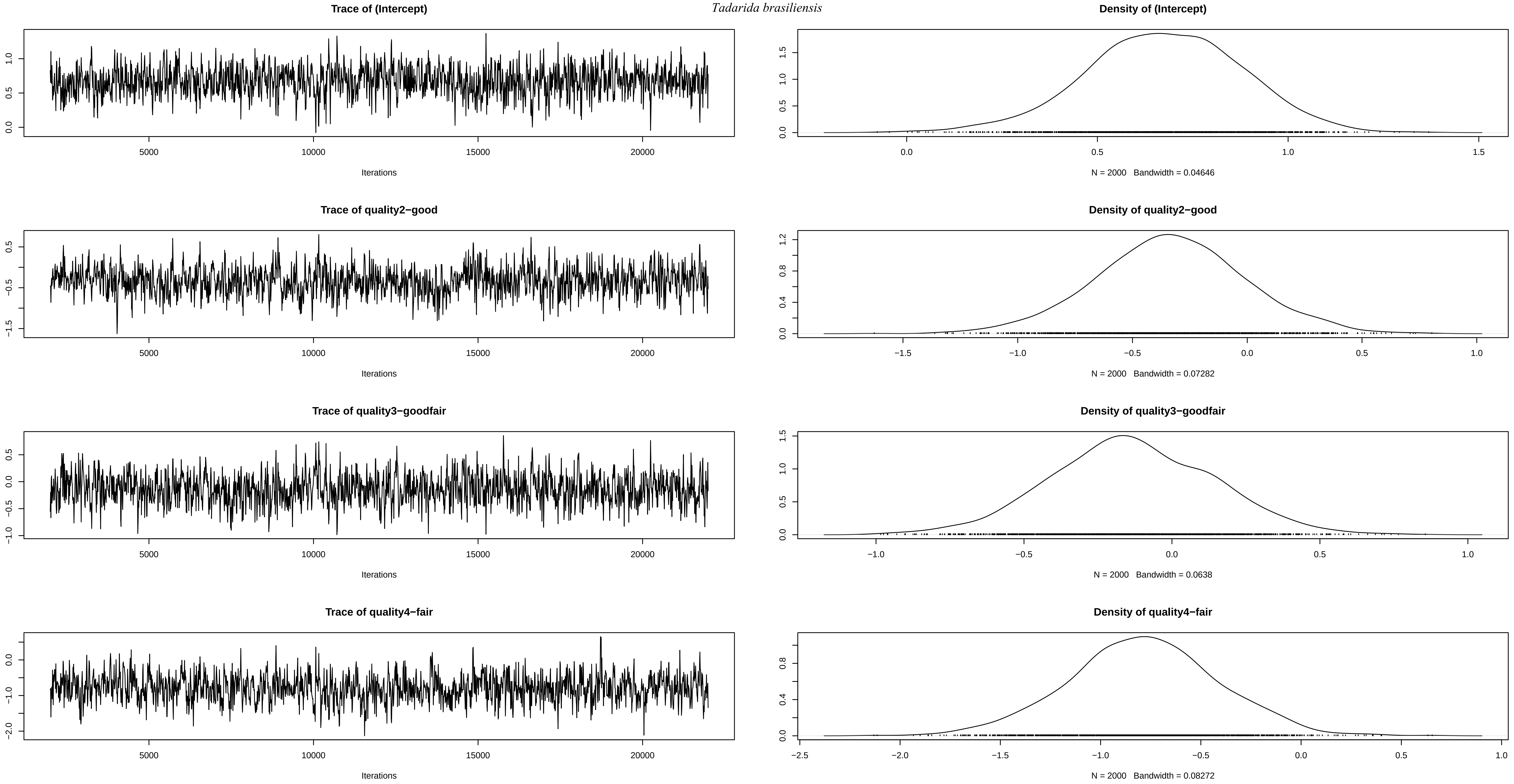

Supplement: Supplementary file 1 [file ECE3-8-667-s001.pdf]
