# Supplementary figures and images for: Separating the effects of water quality and urbanization on temperate insectivorous bats at the landscape scale
Source: Ecol Evol. 2017 Dec 3;8(1):667–78. doi: 10.1002/ece3.3693 (PMC5756845; doi:10.1002/ece3.3693)

*Eptesicus fuscus*

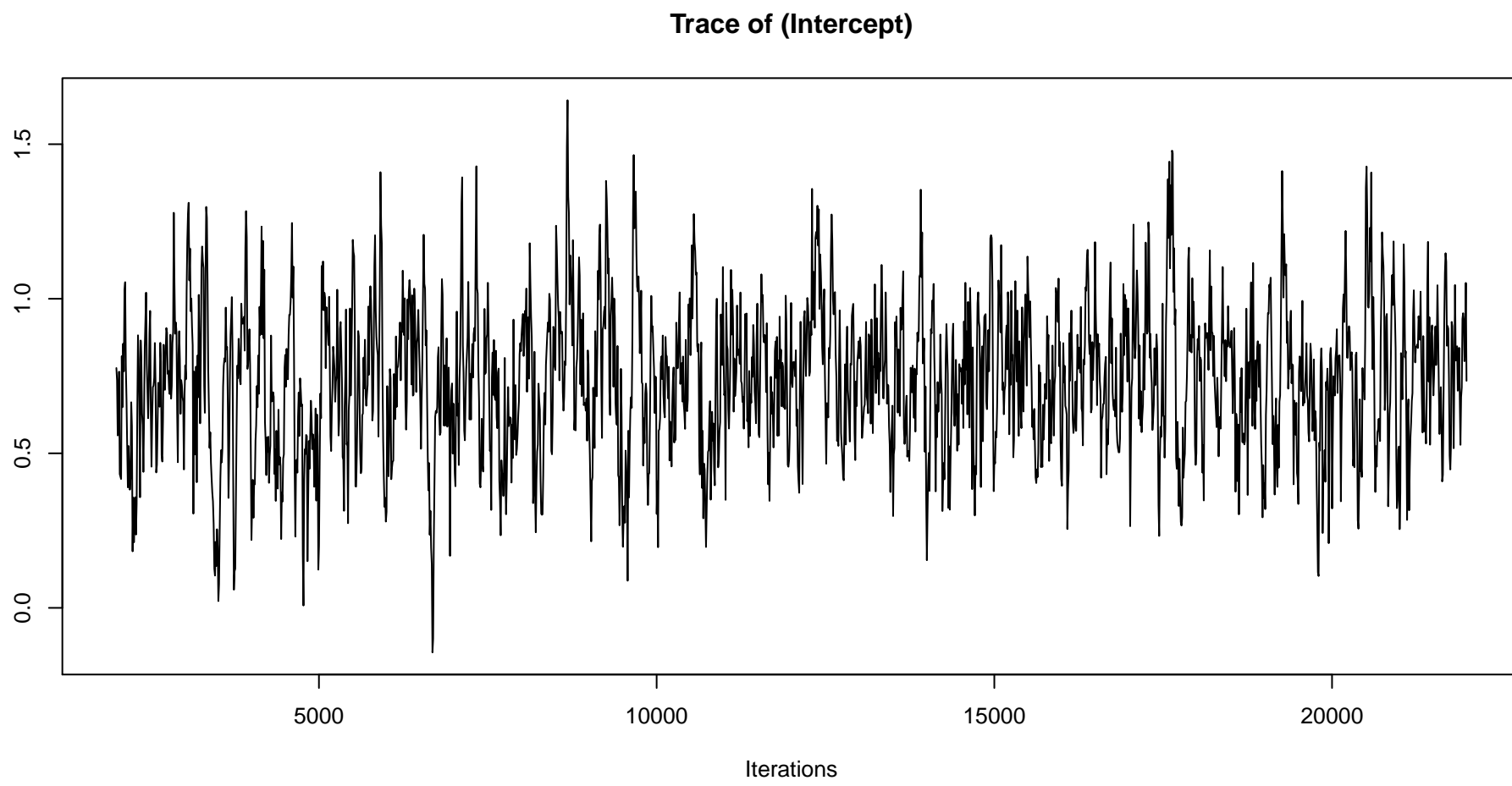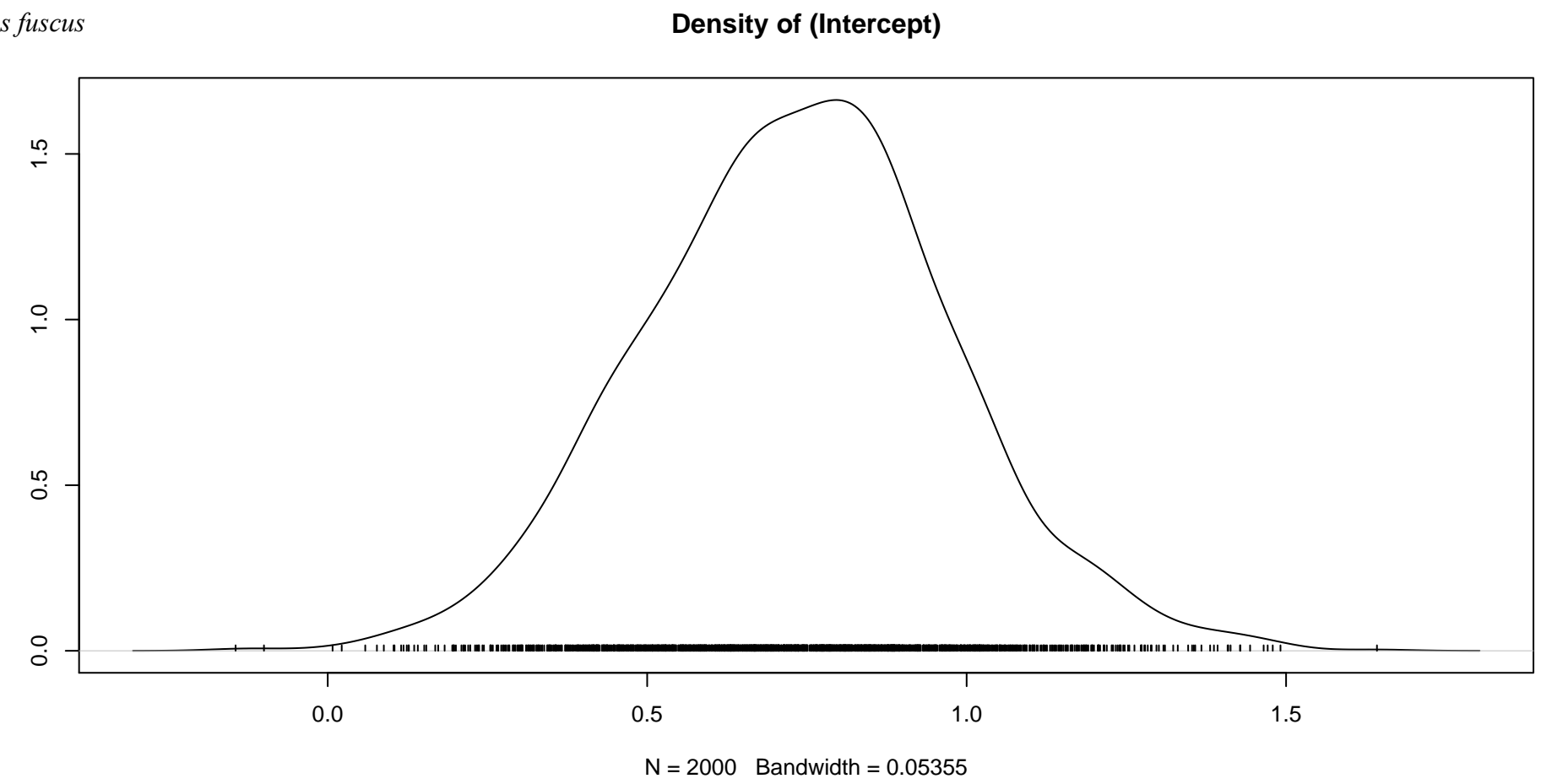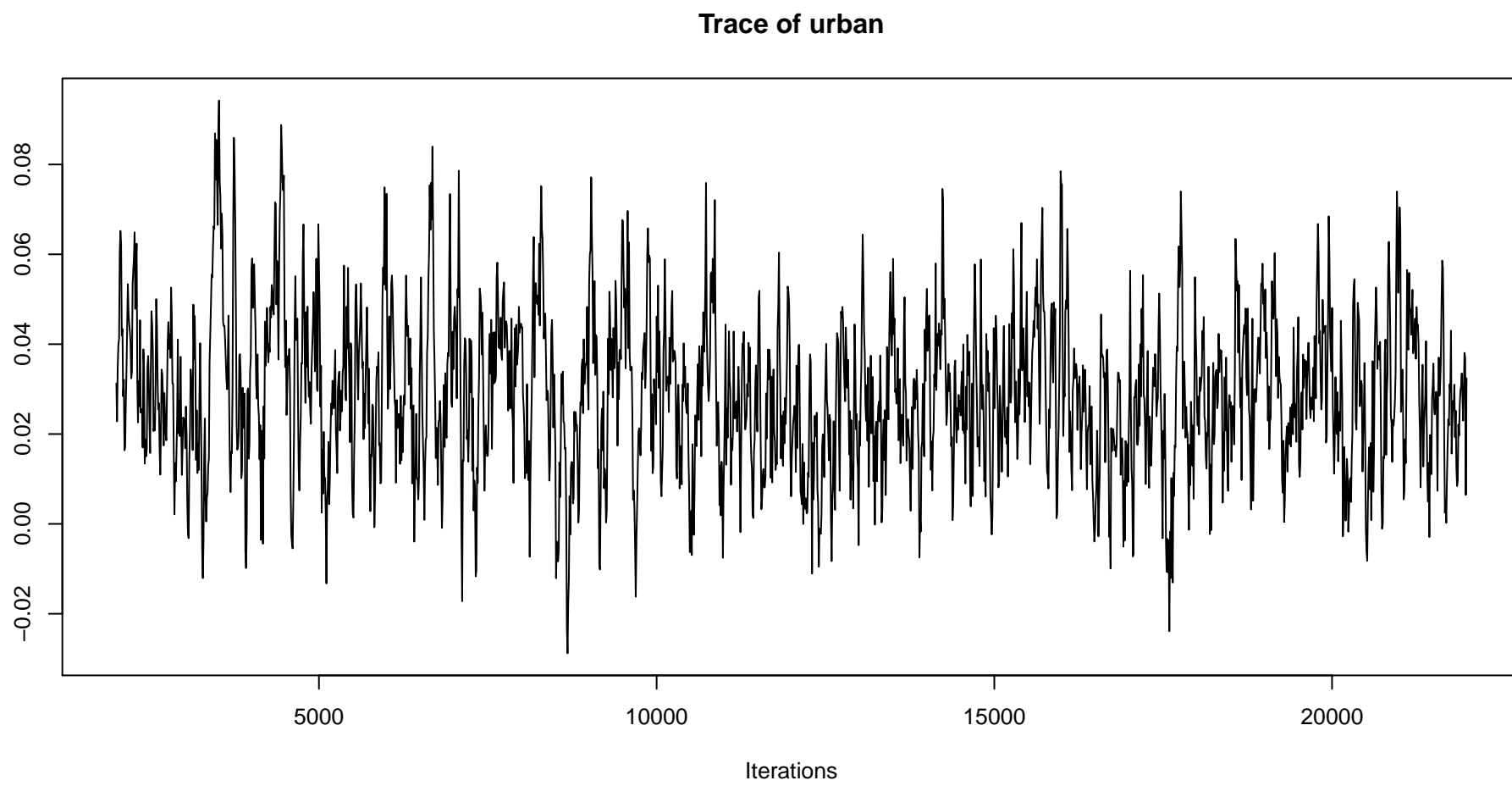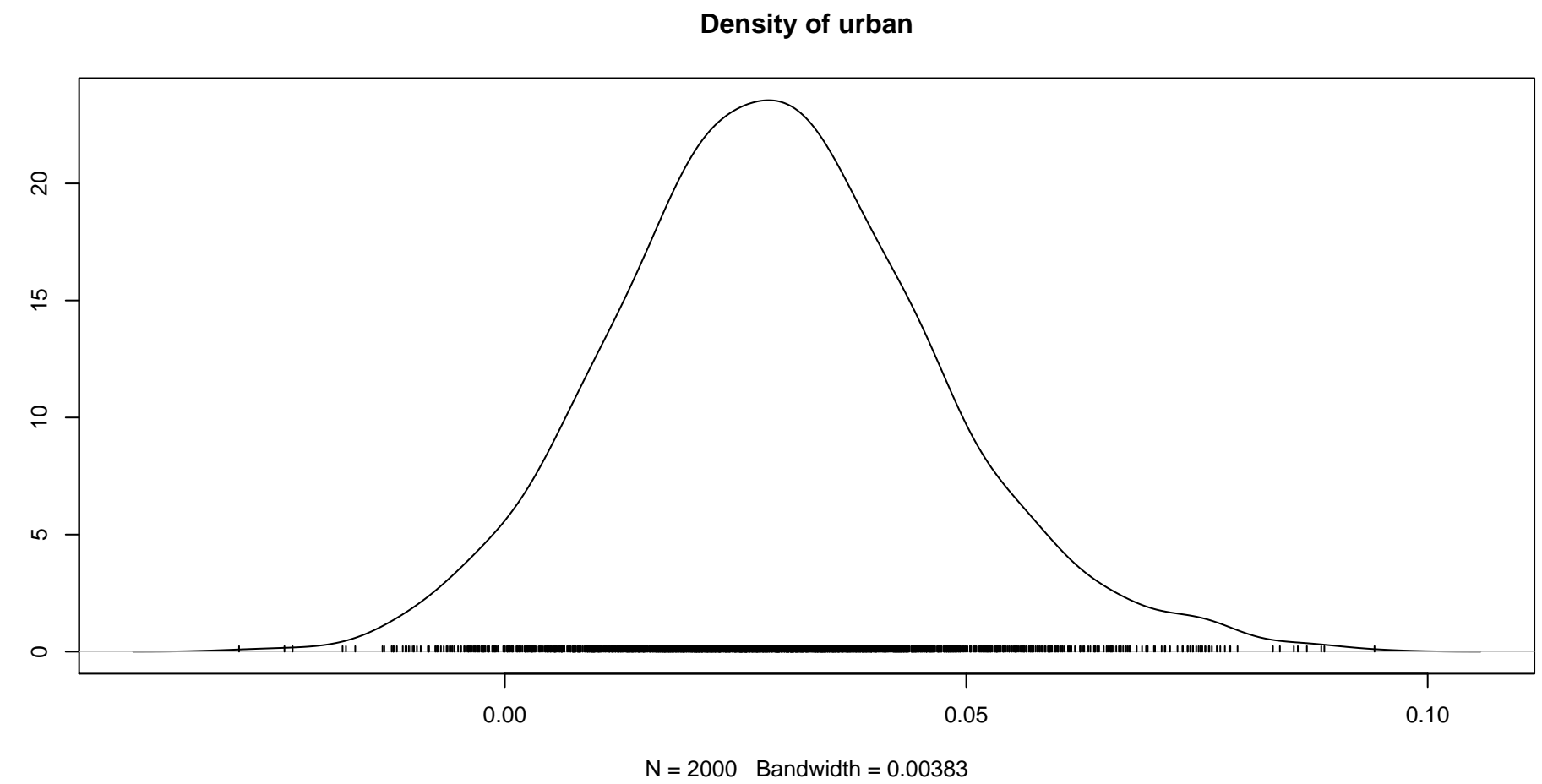

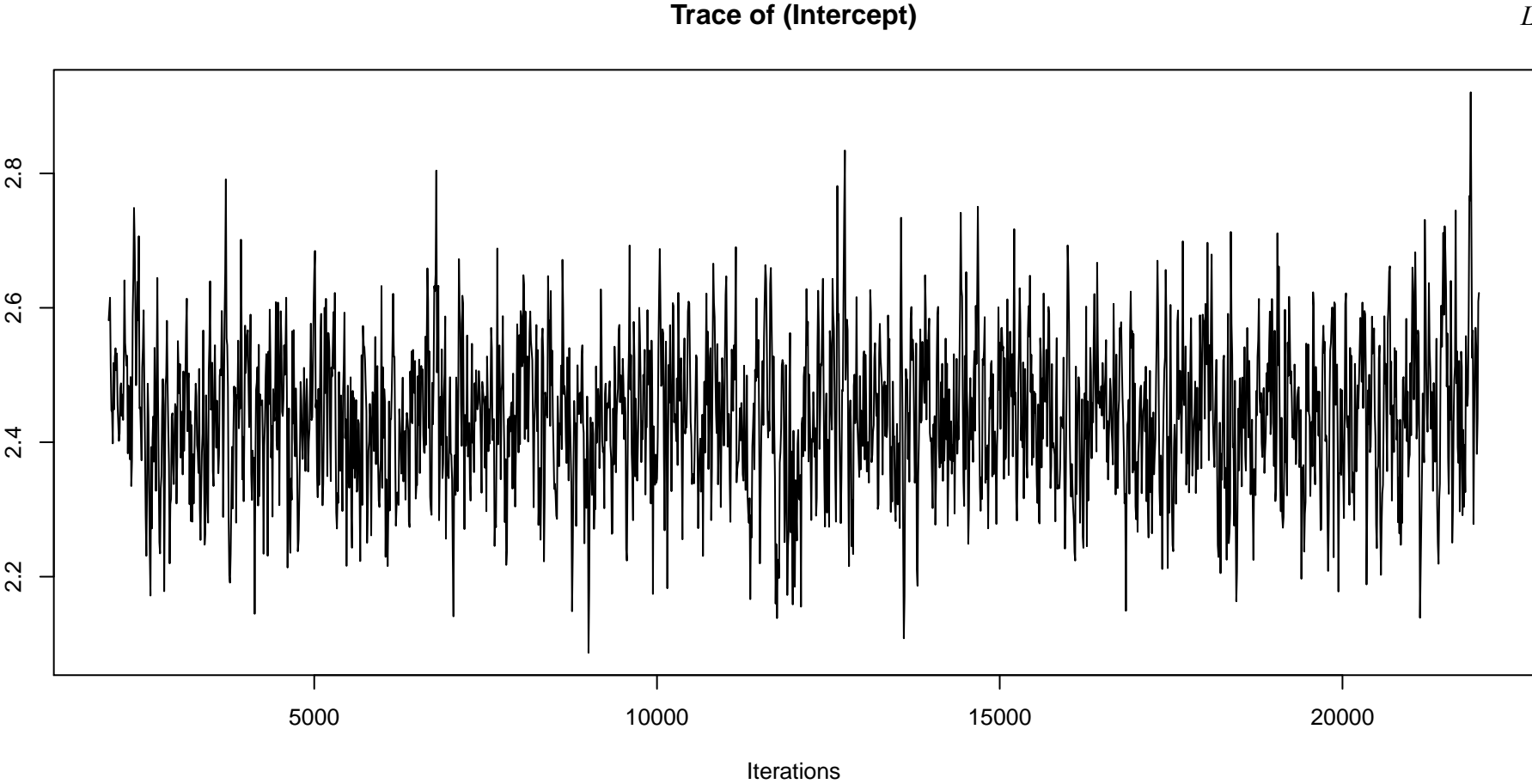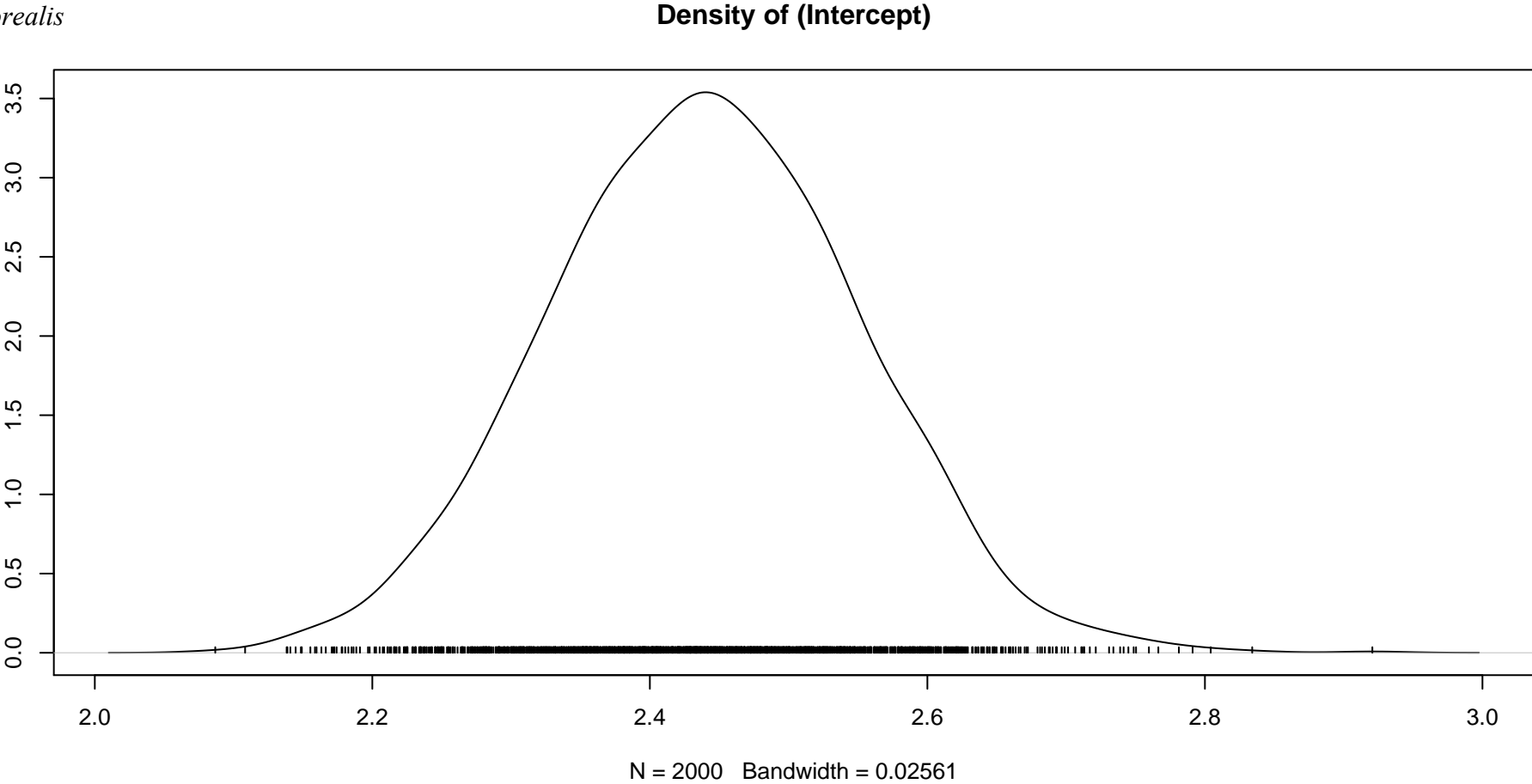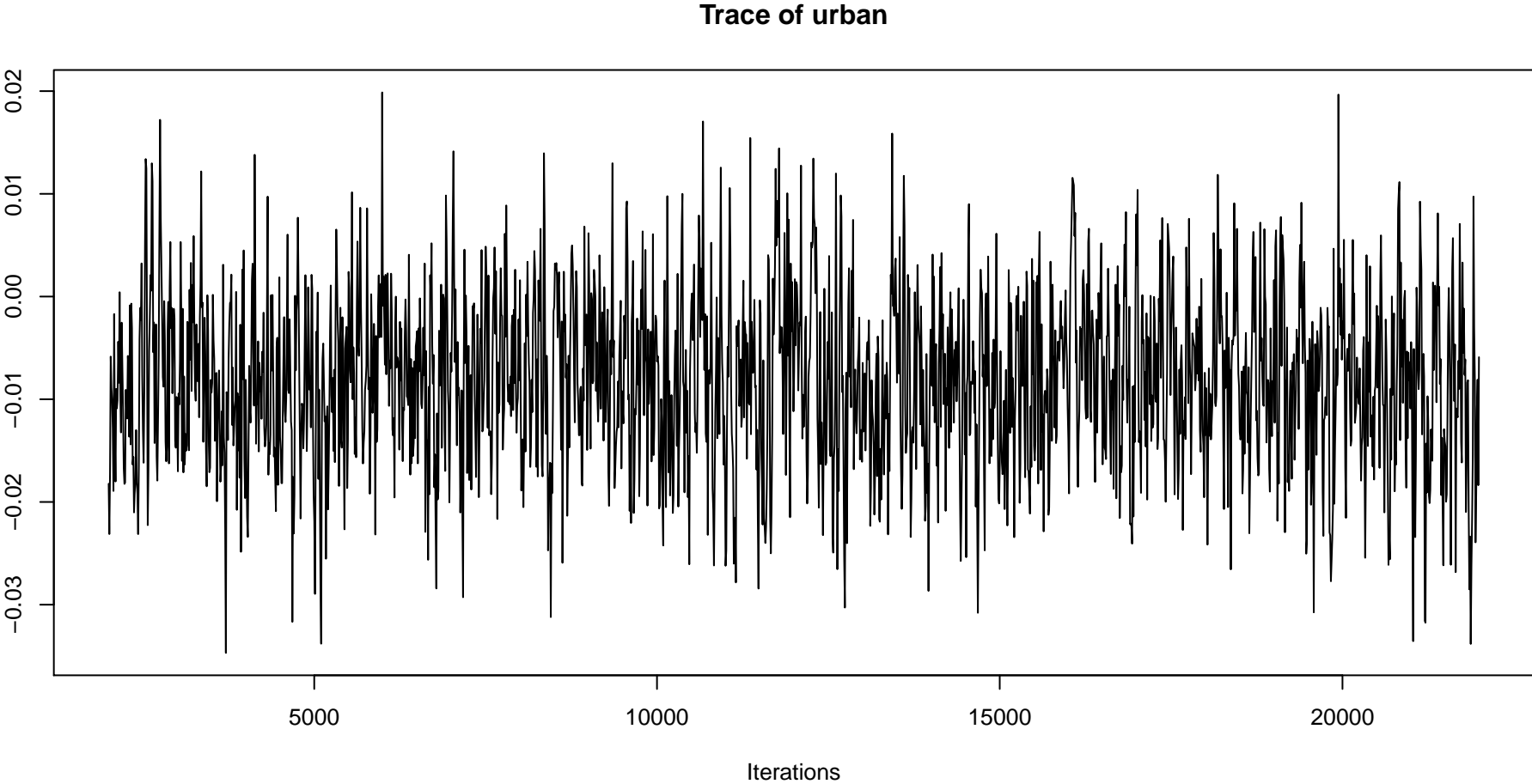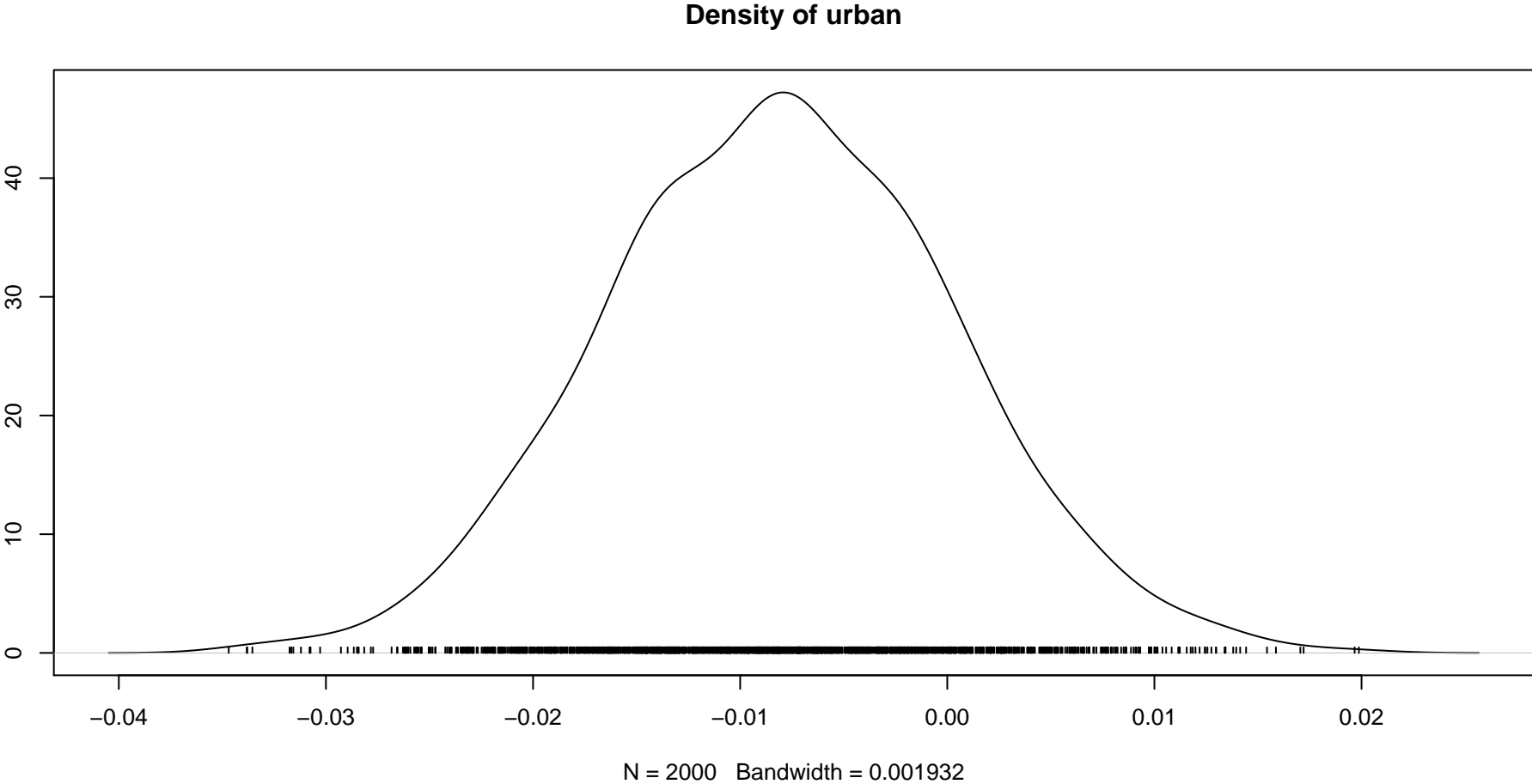

*Lasiurus cinereus*

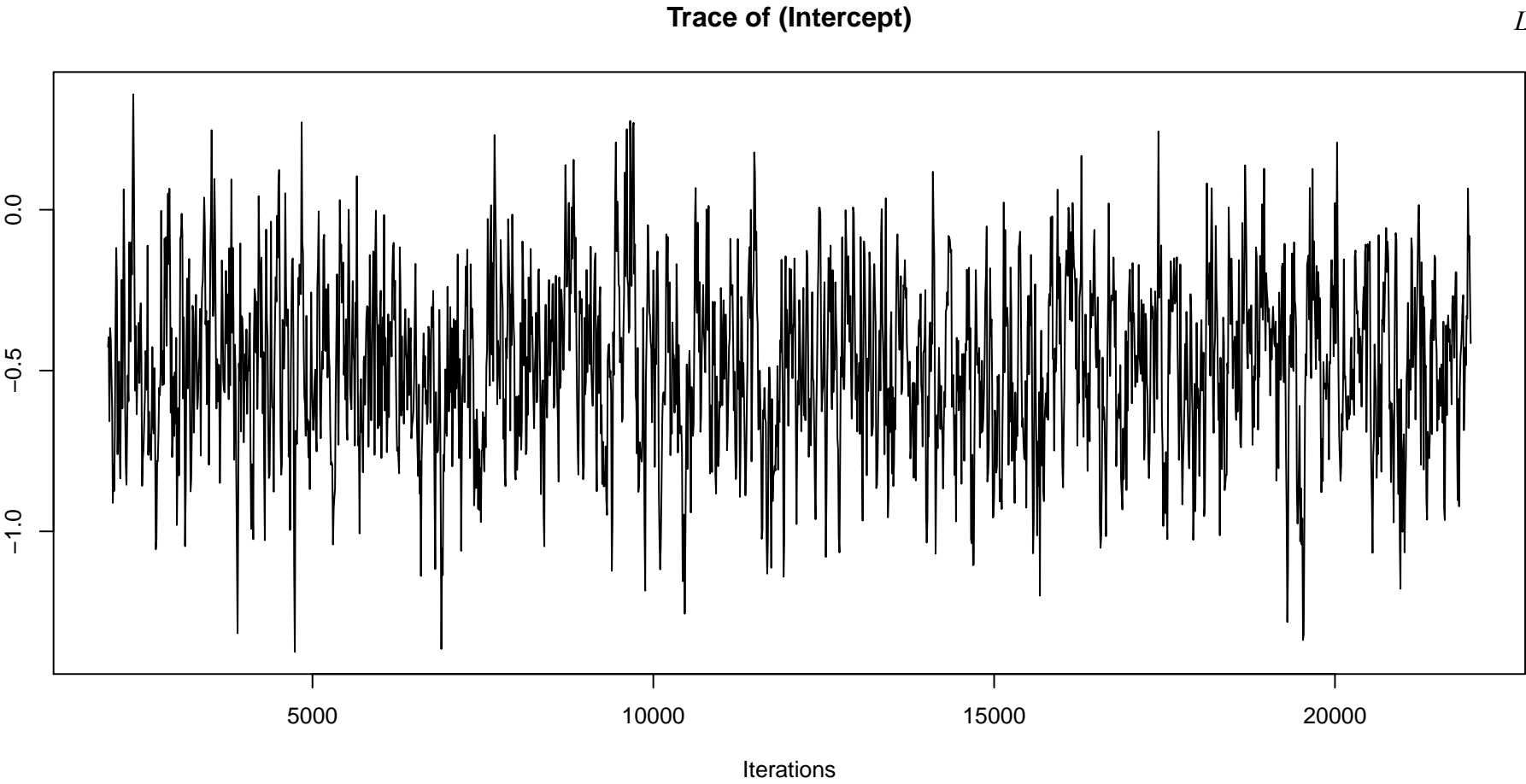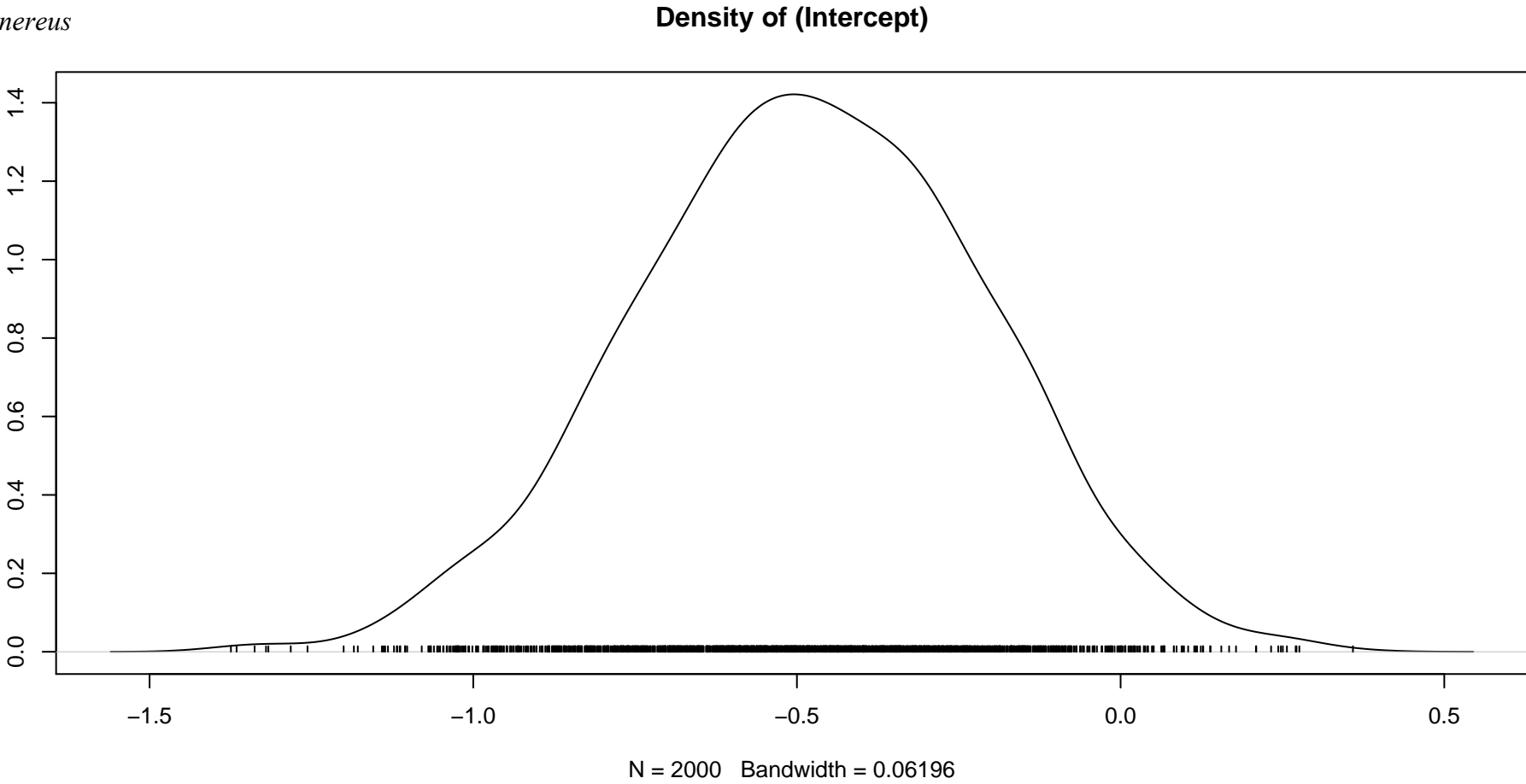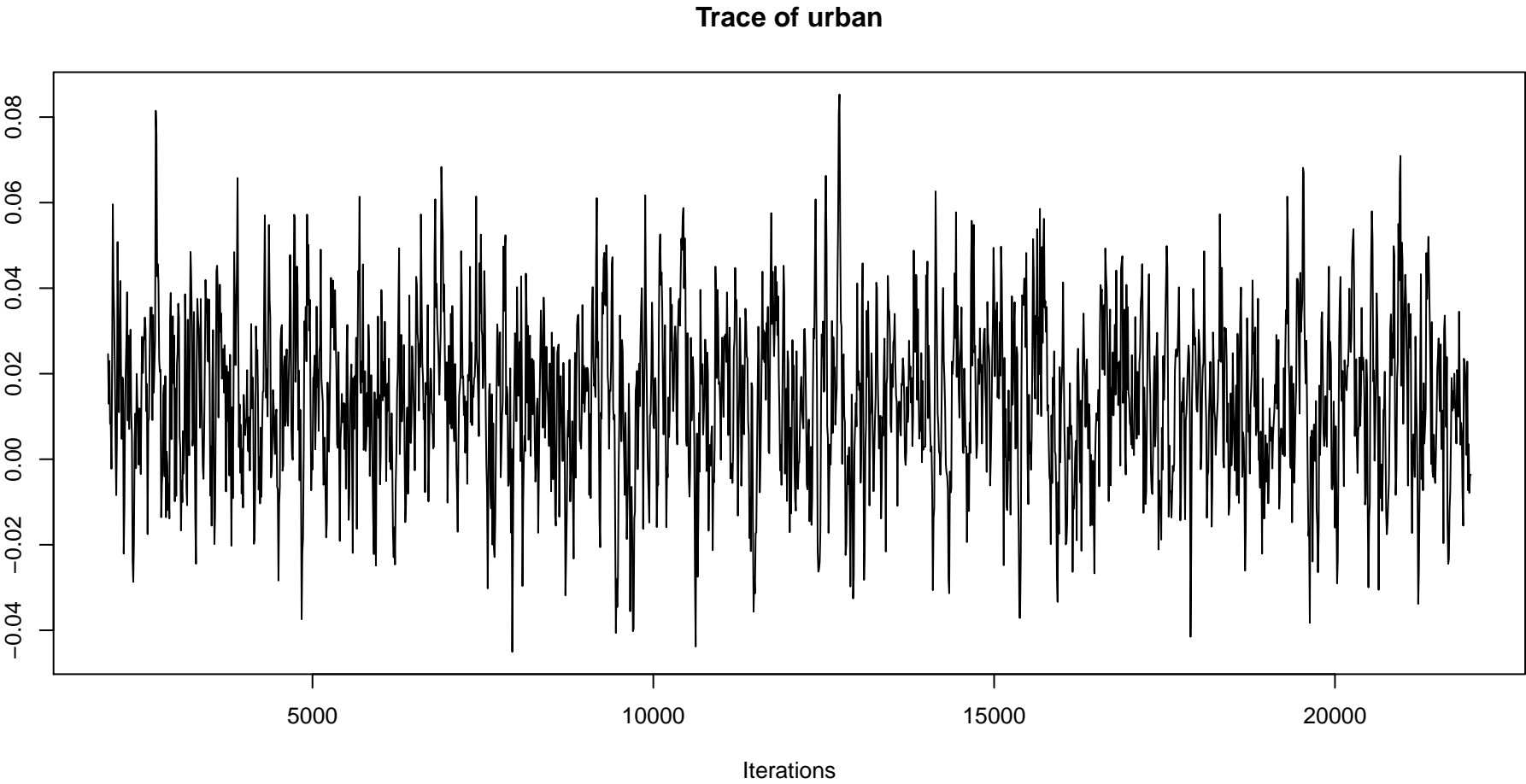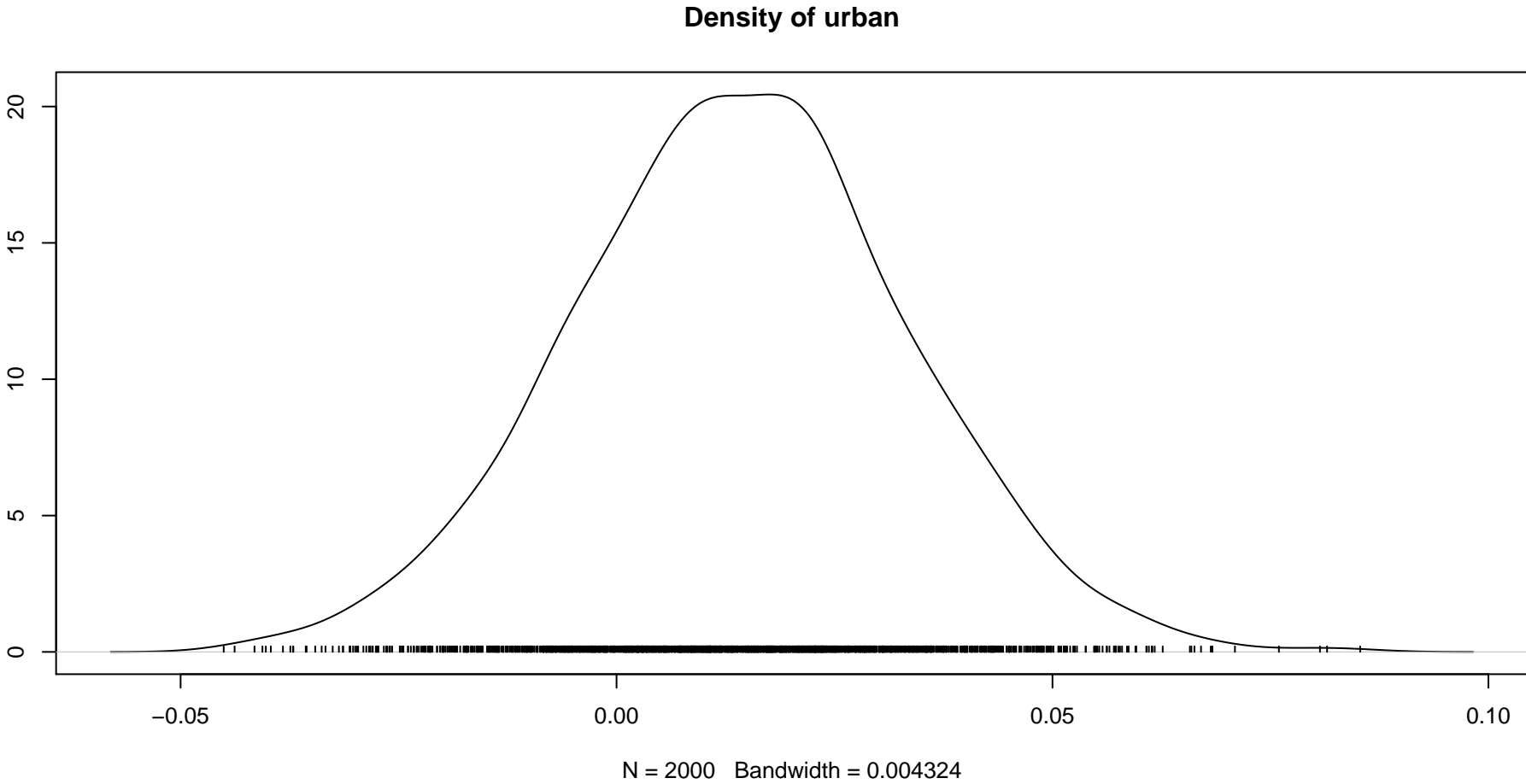

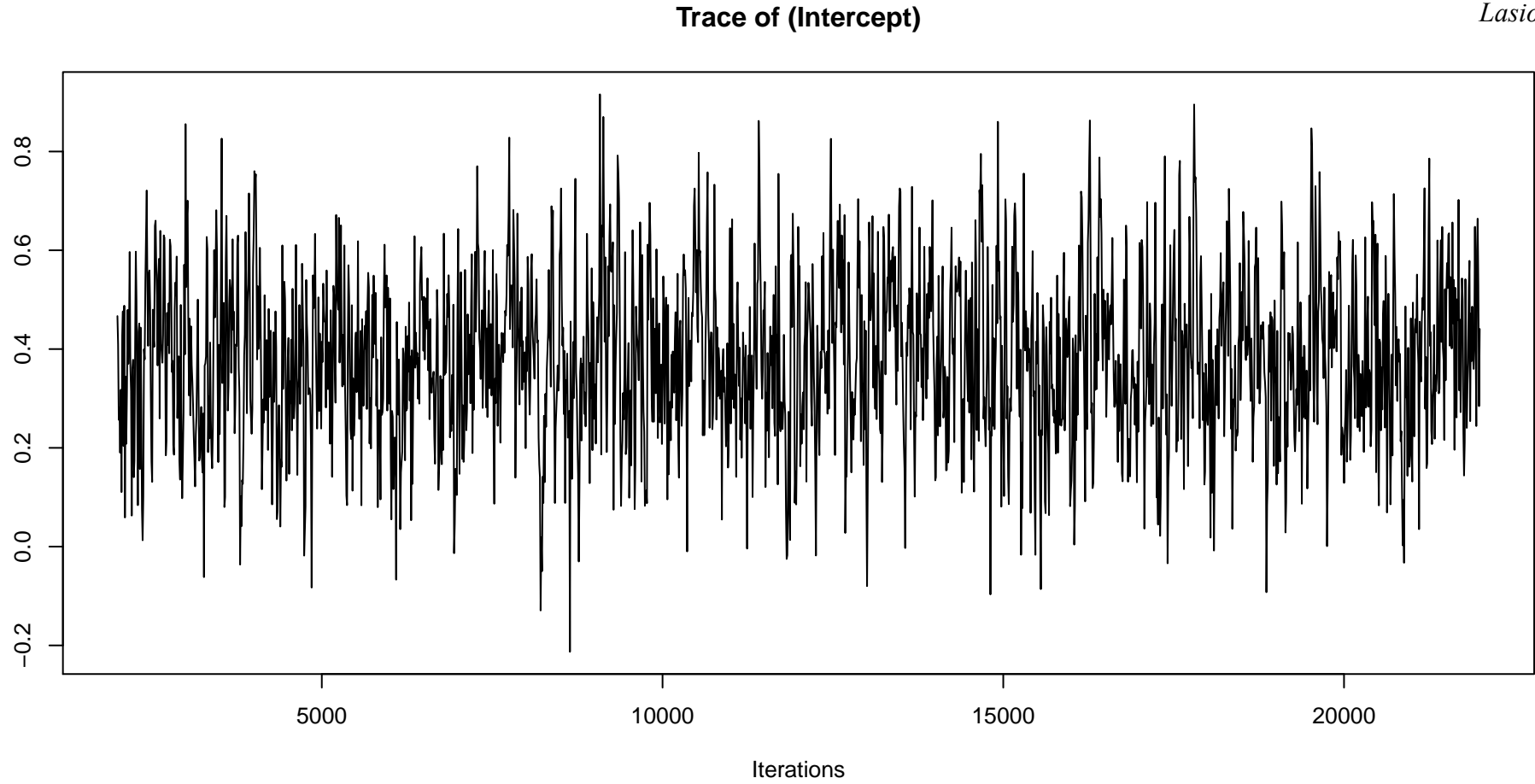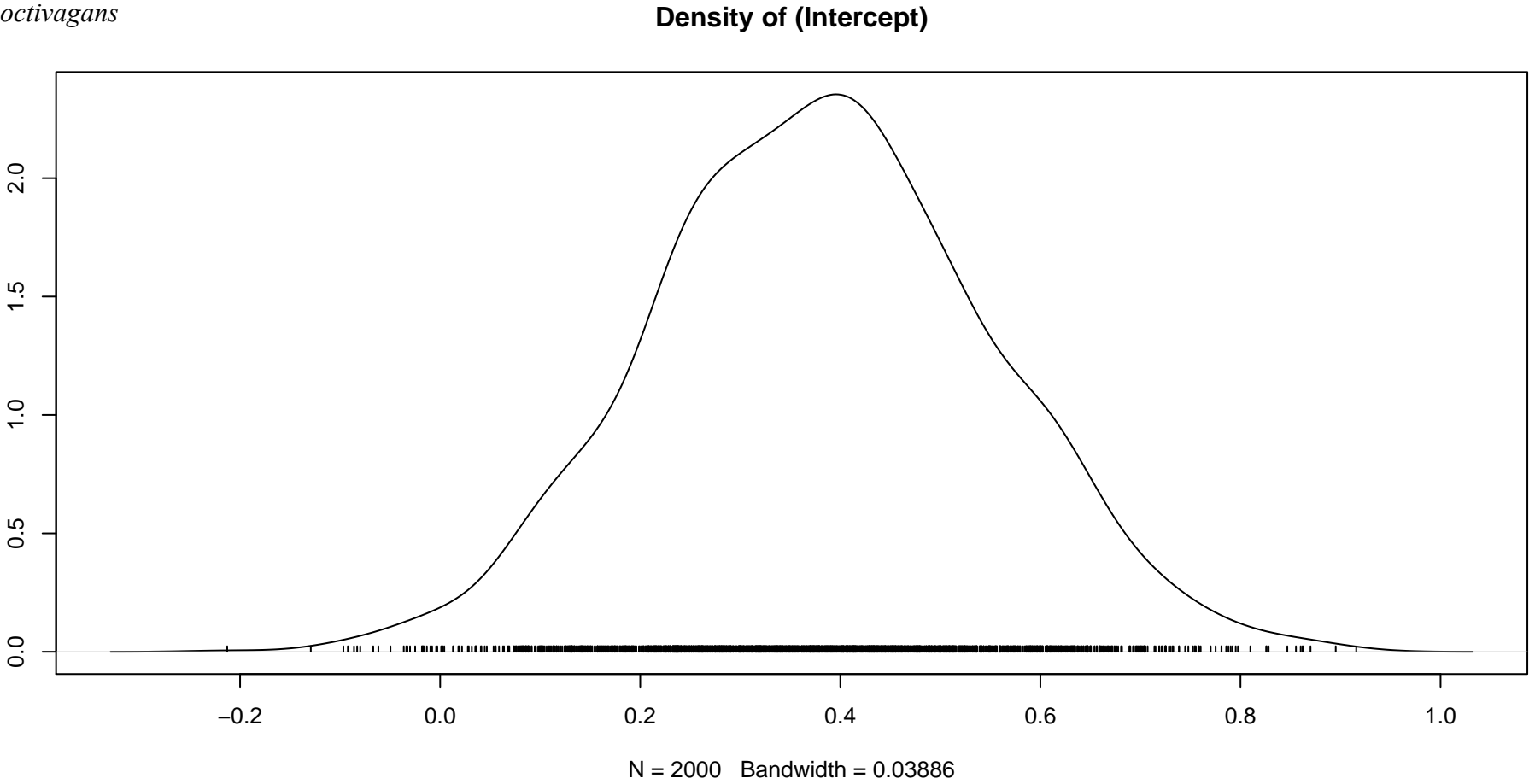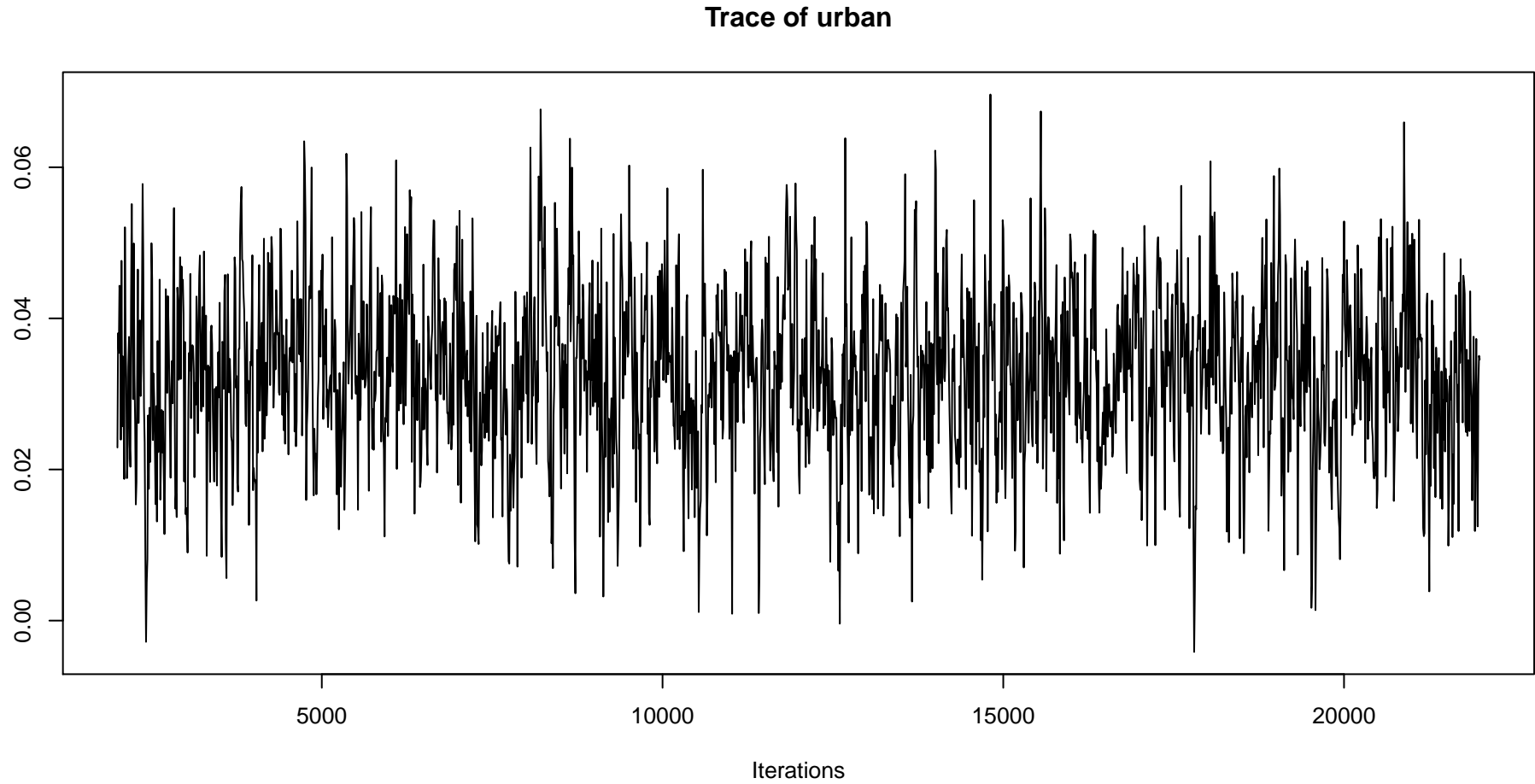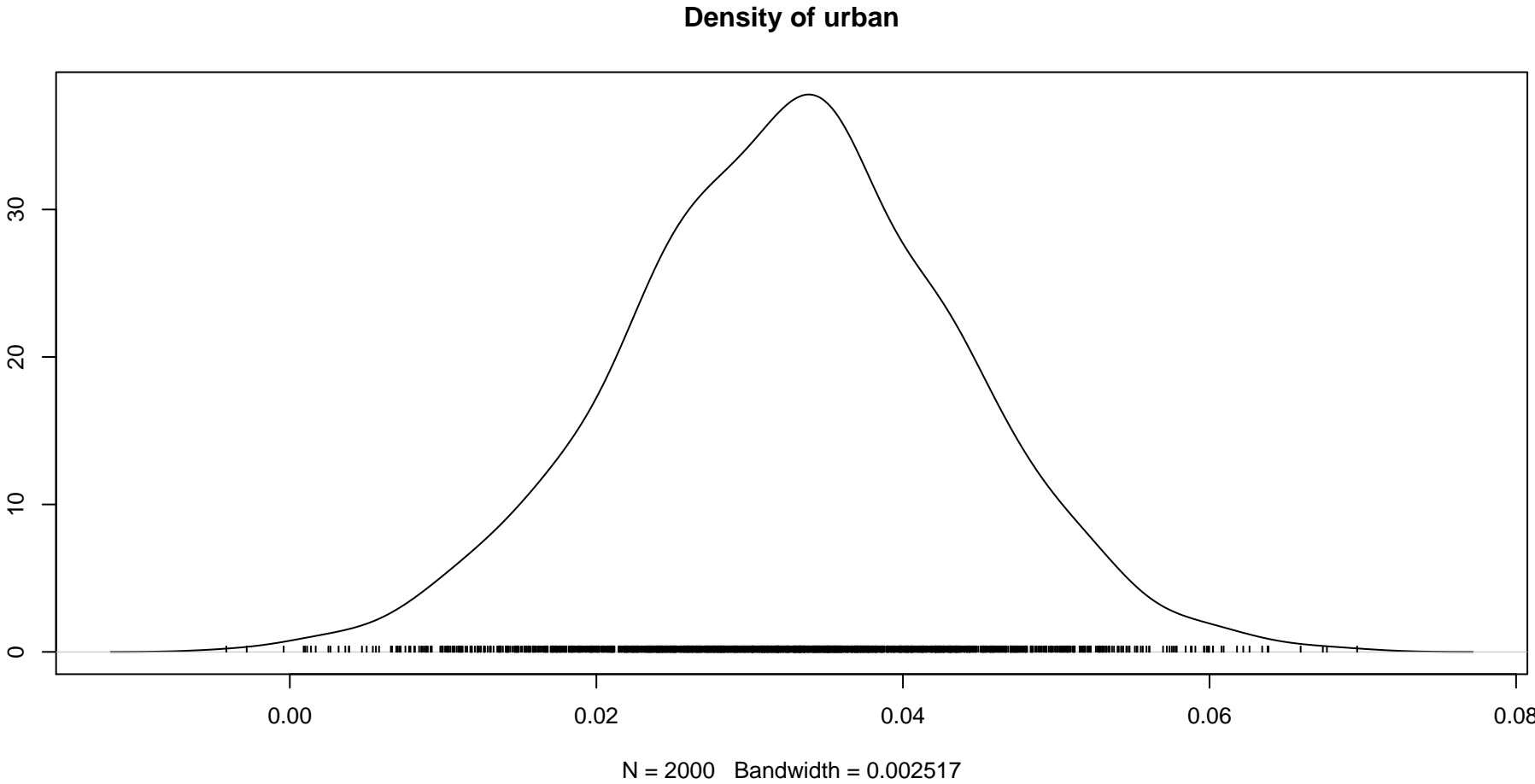

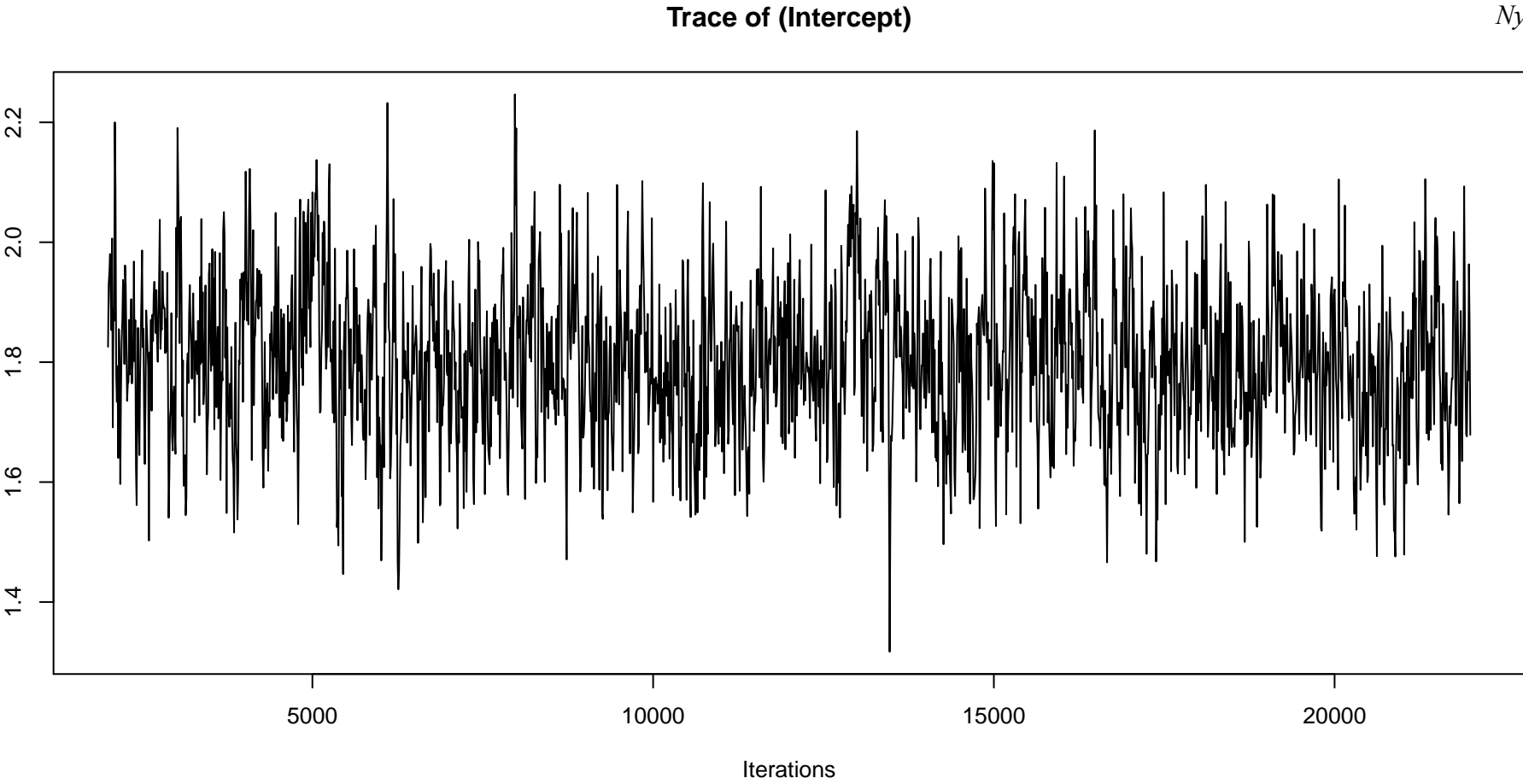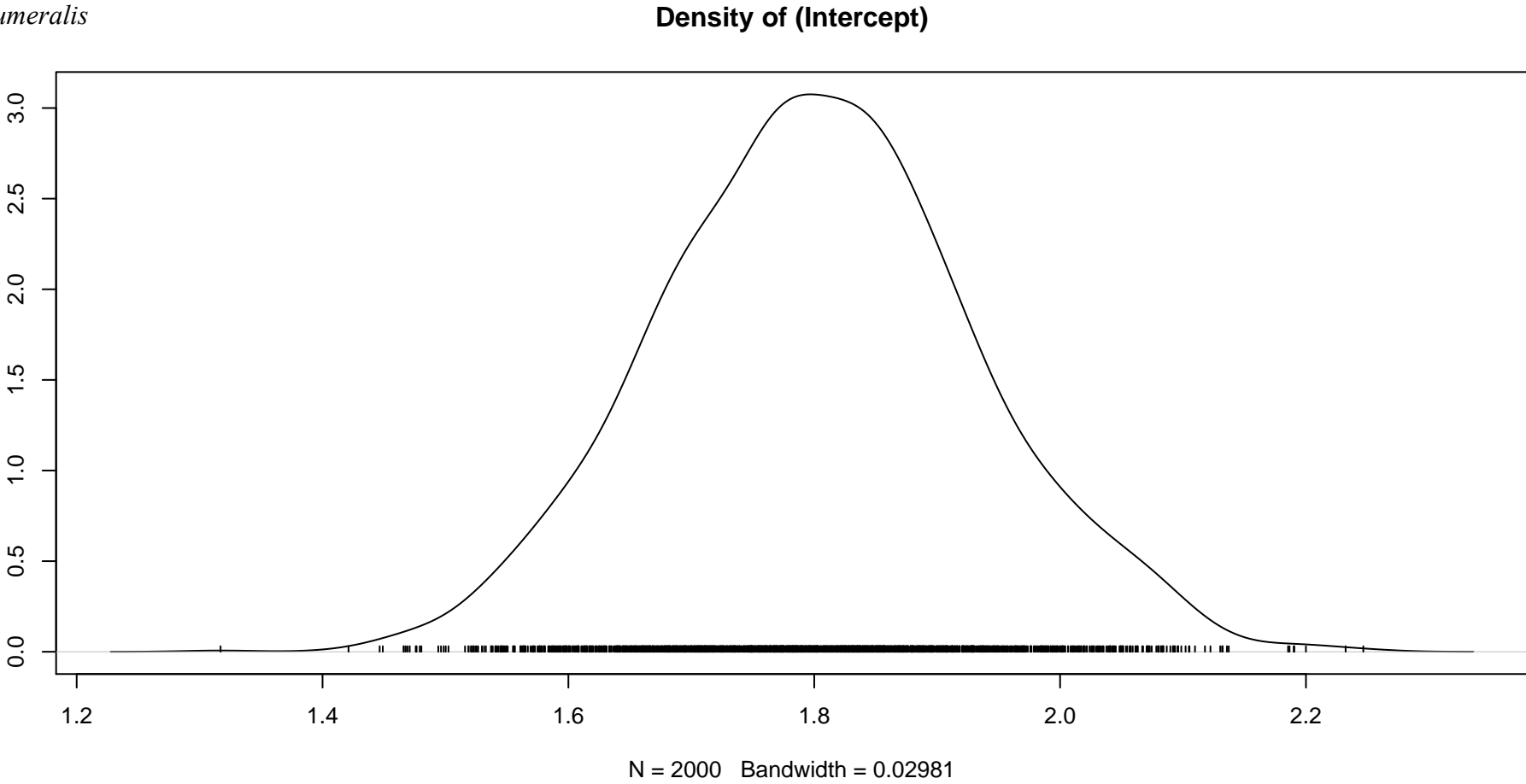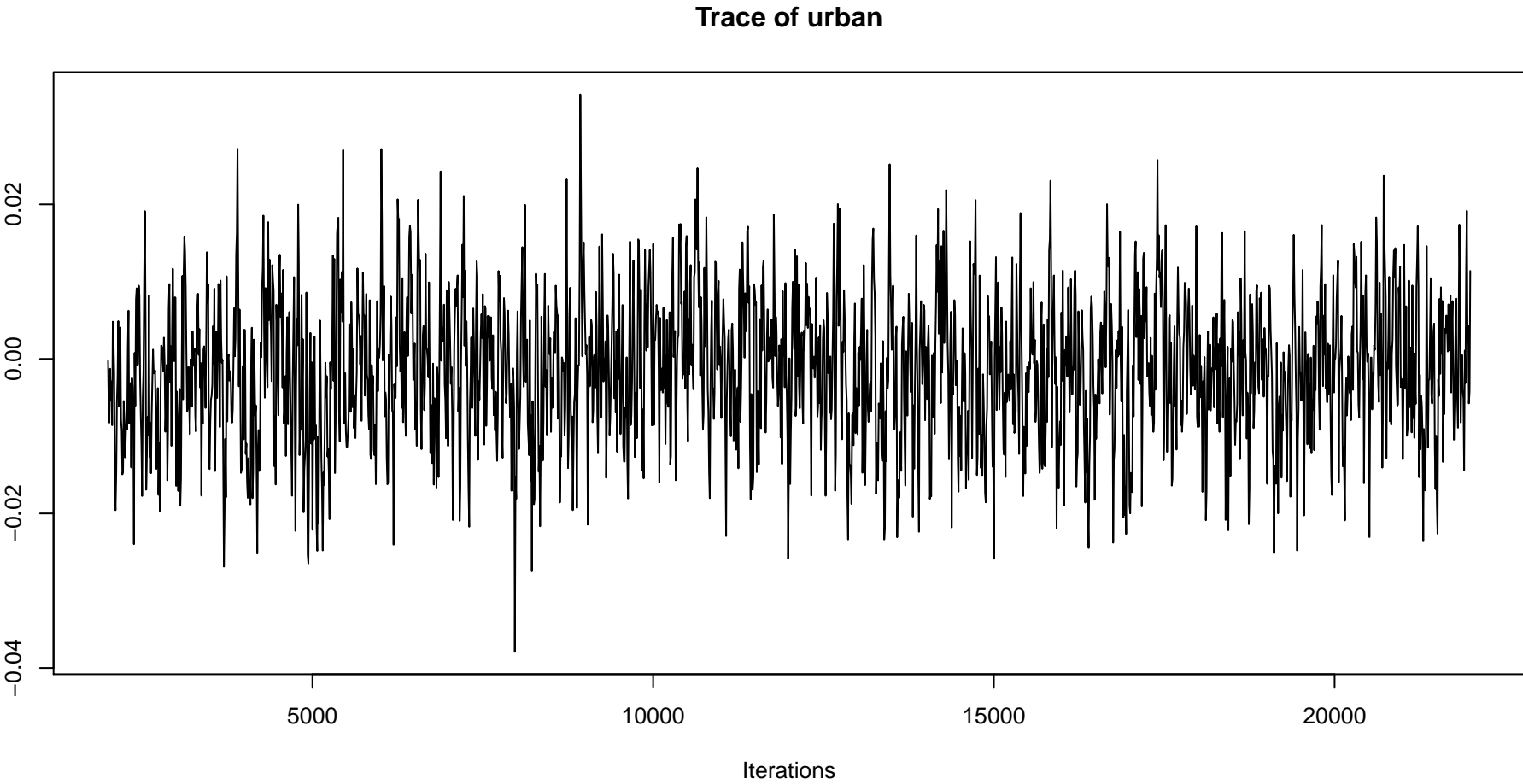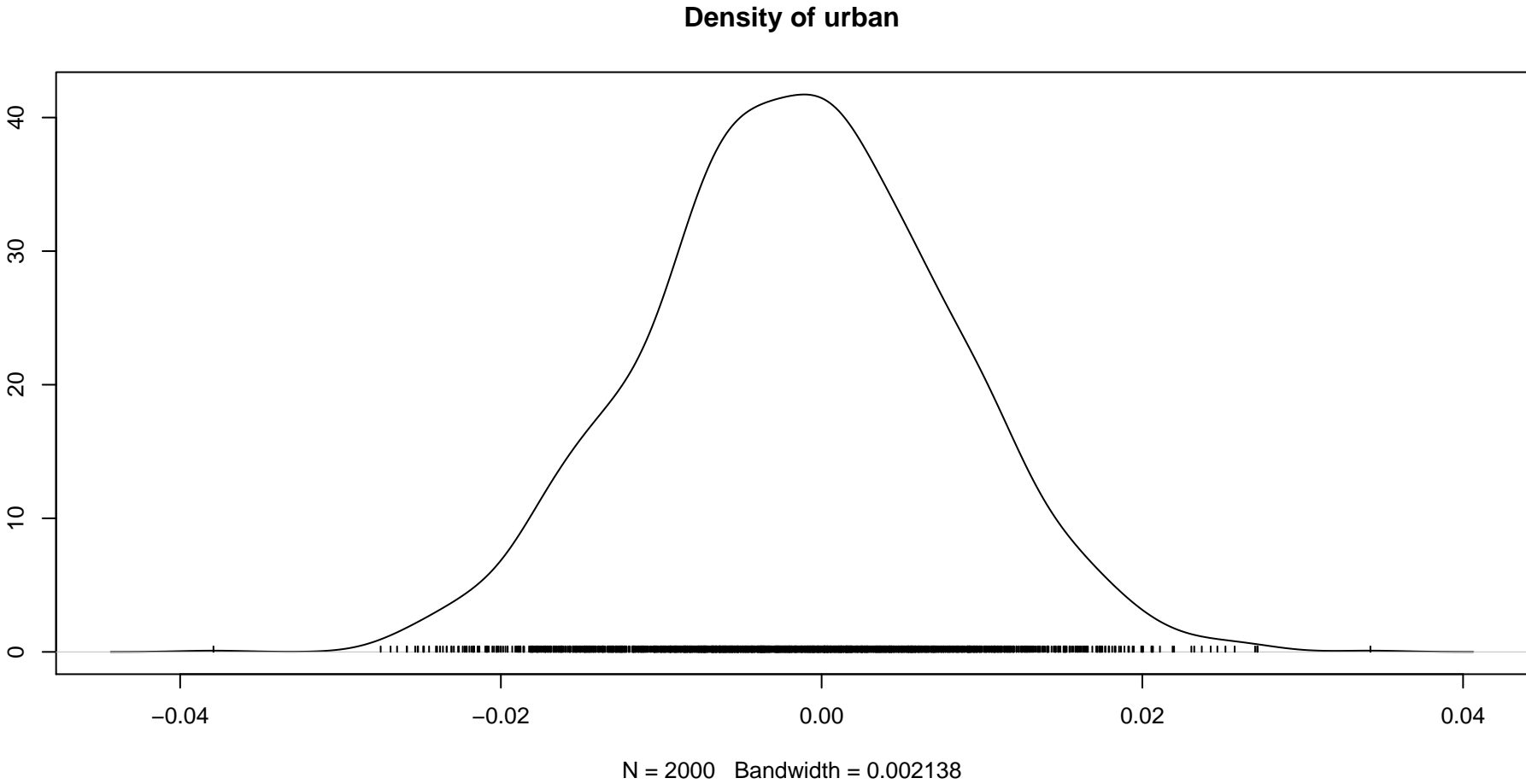

*Perimyotis subflavus*

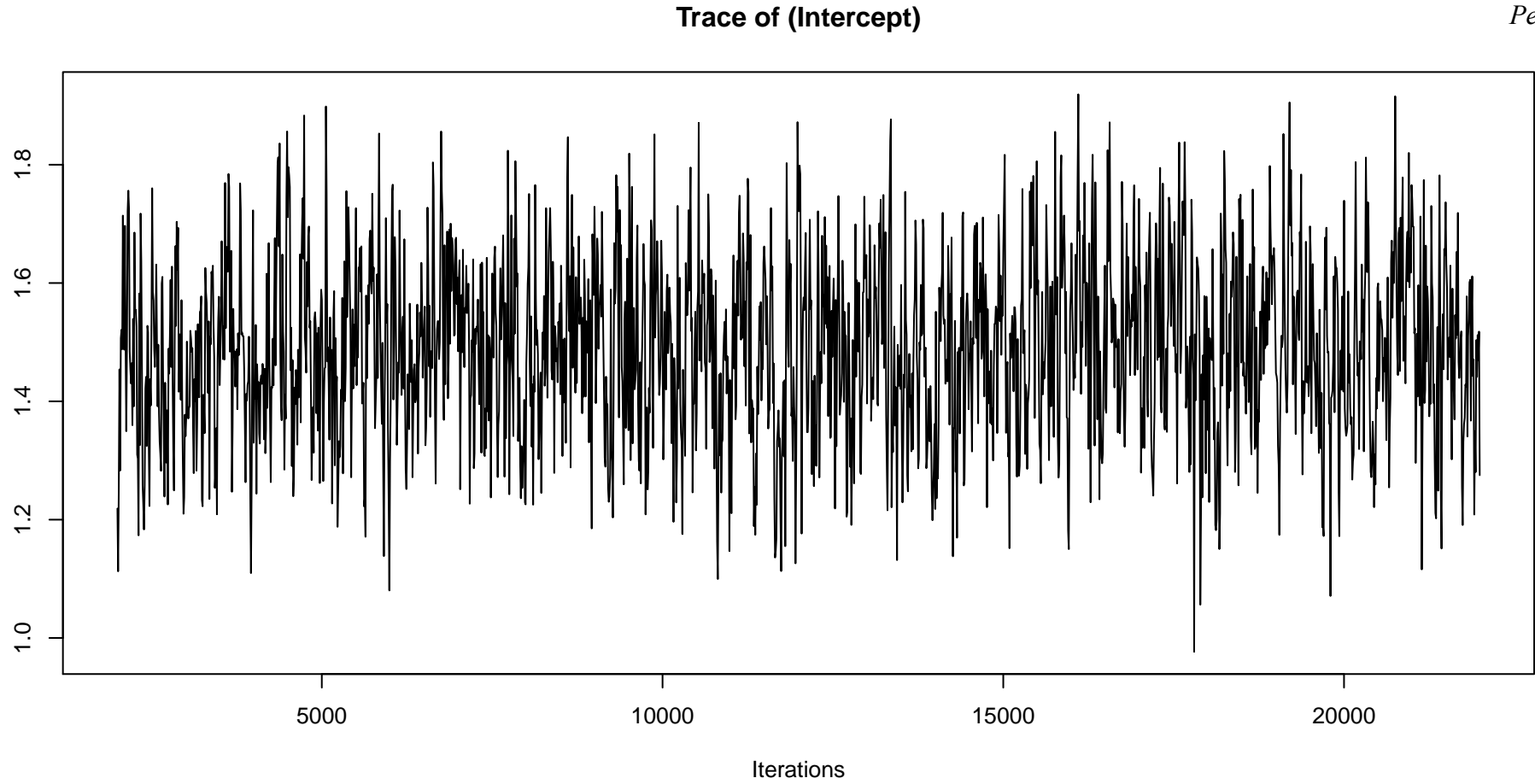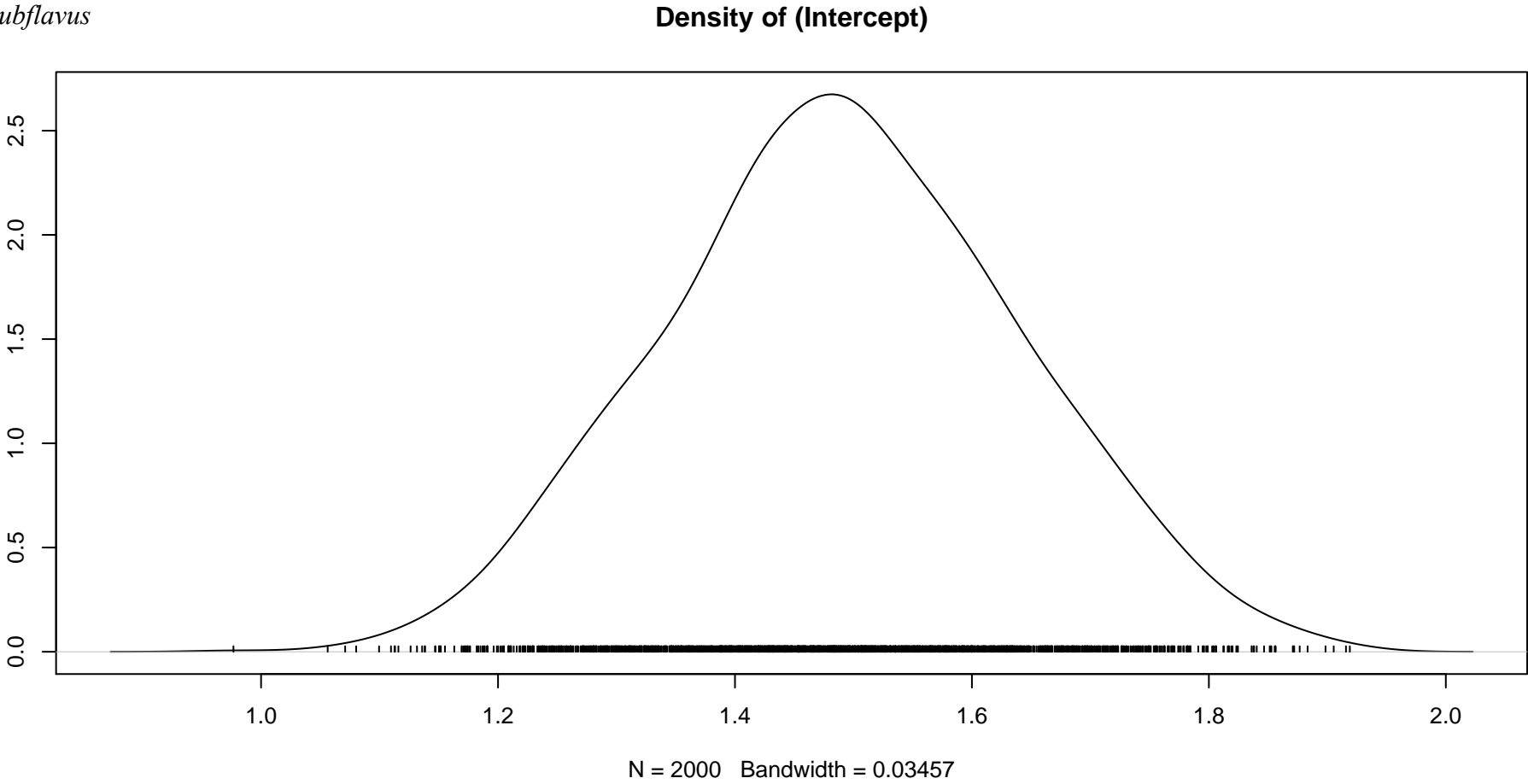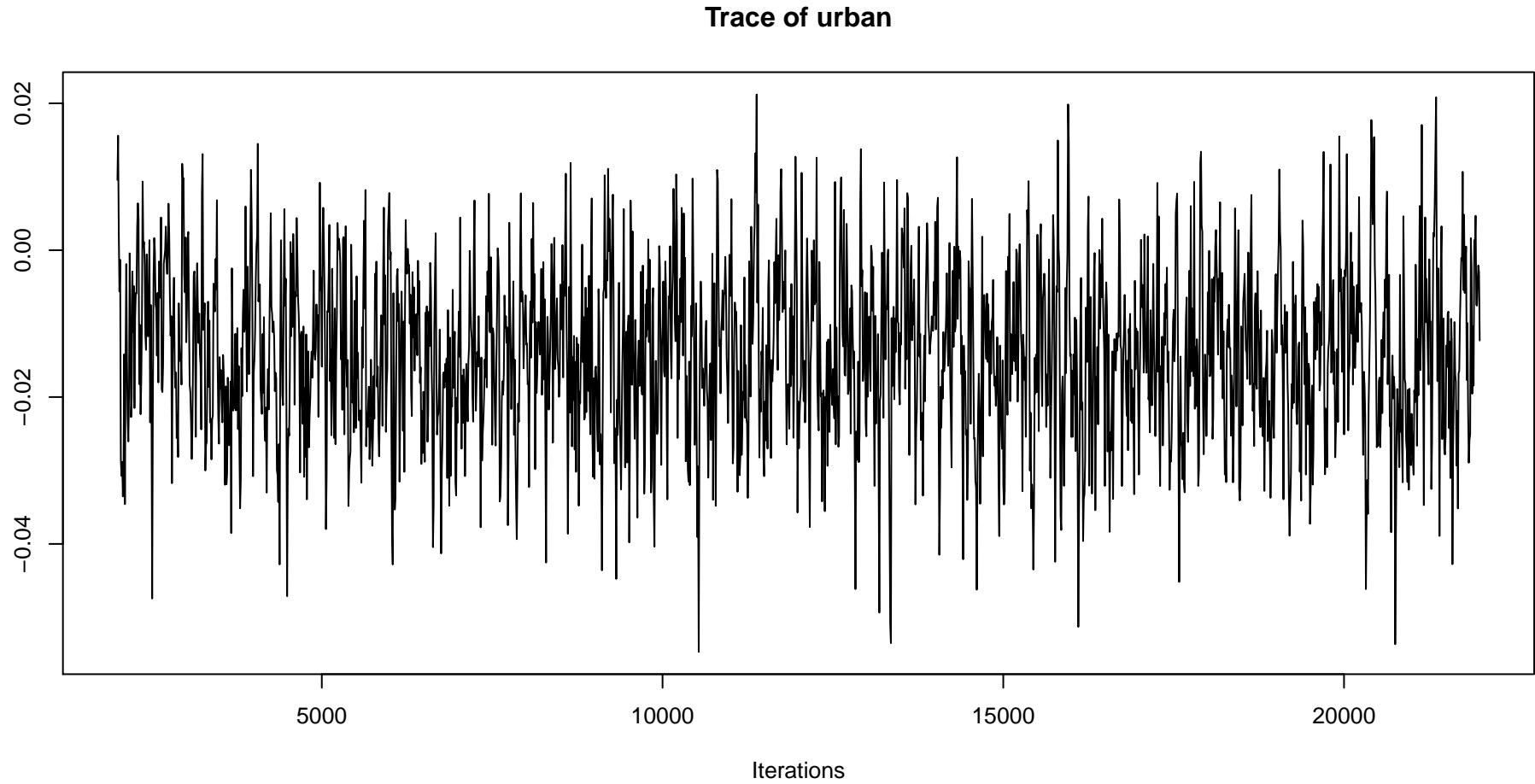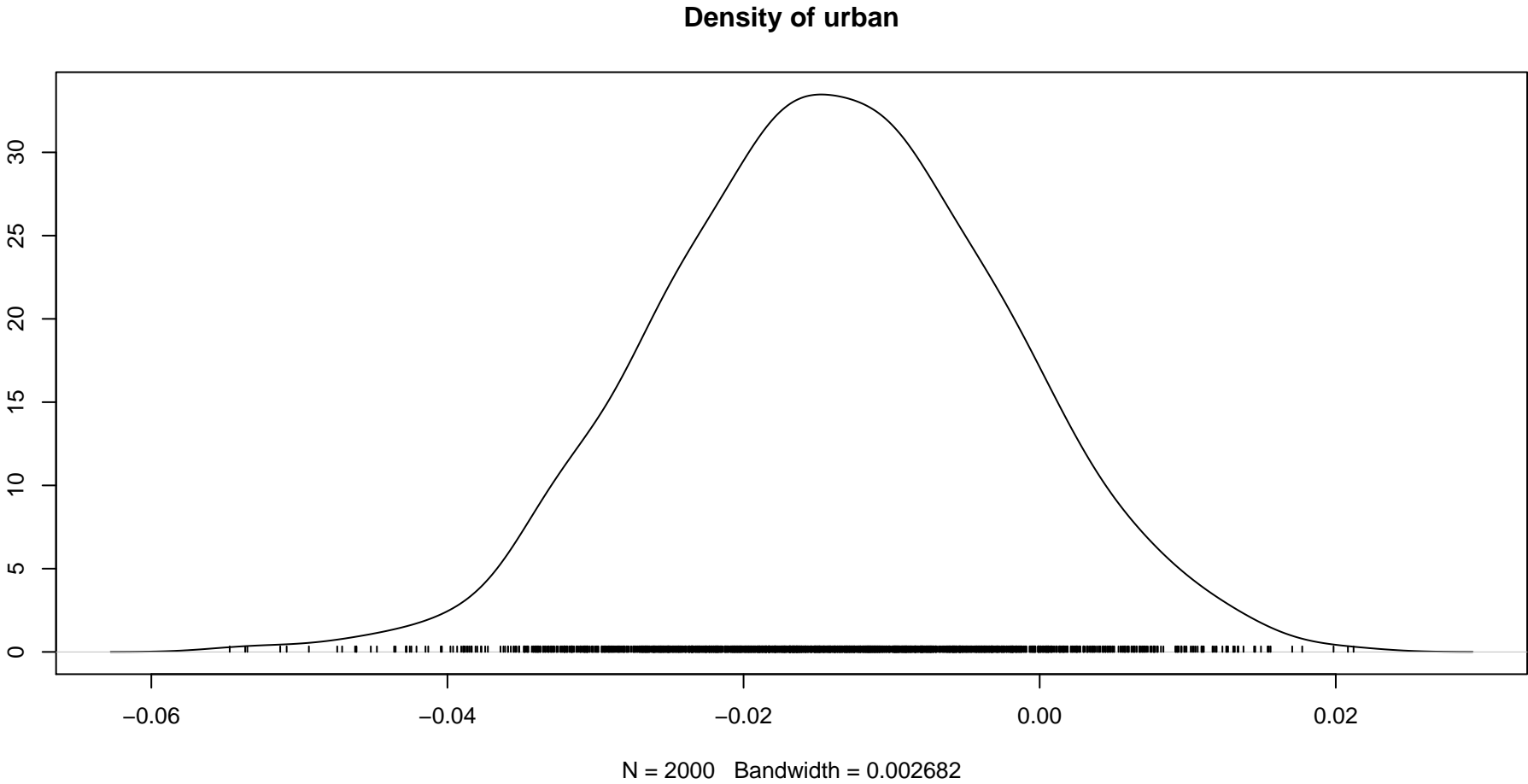

*Tadarida brasiliensis*

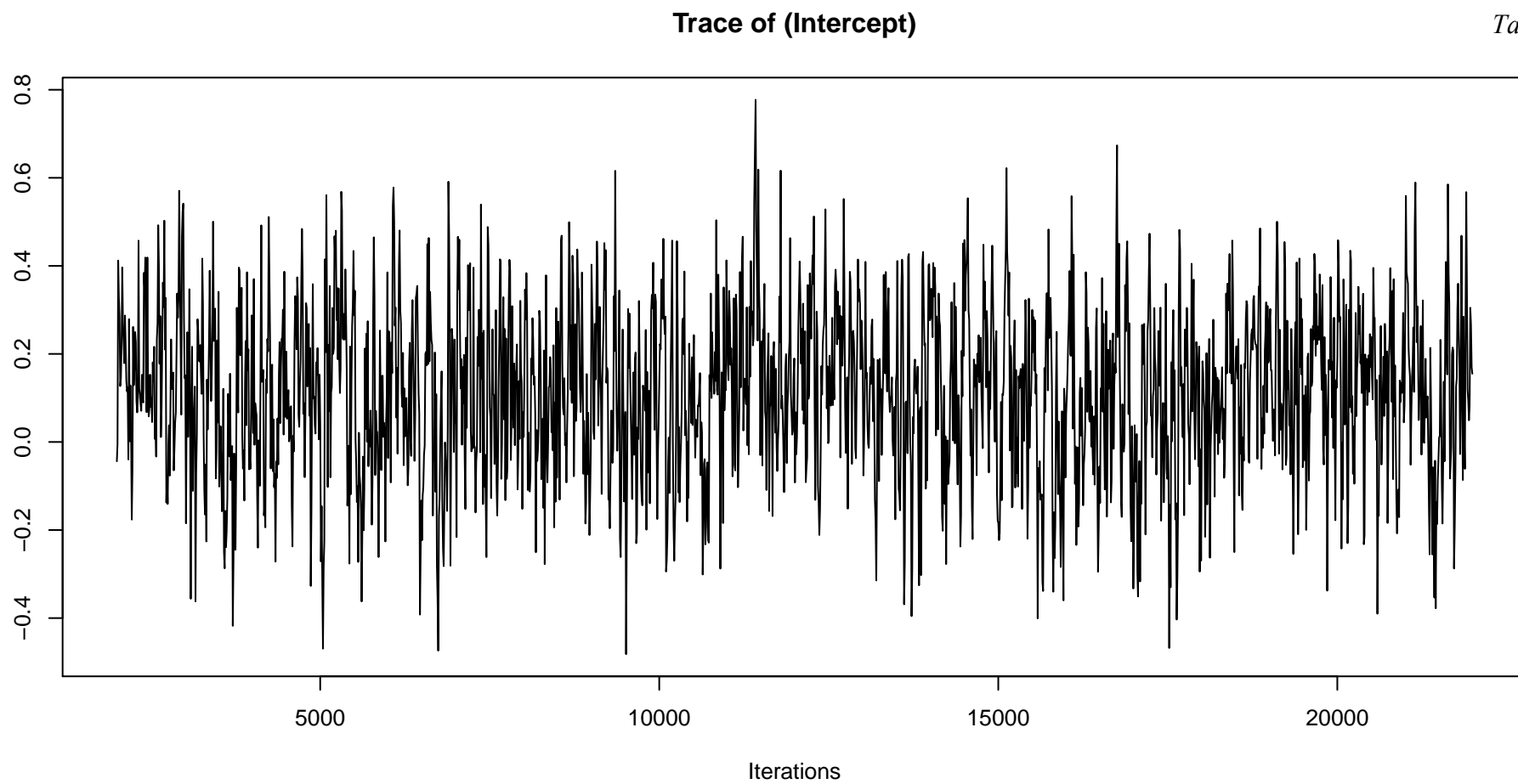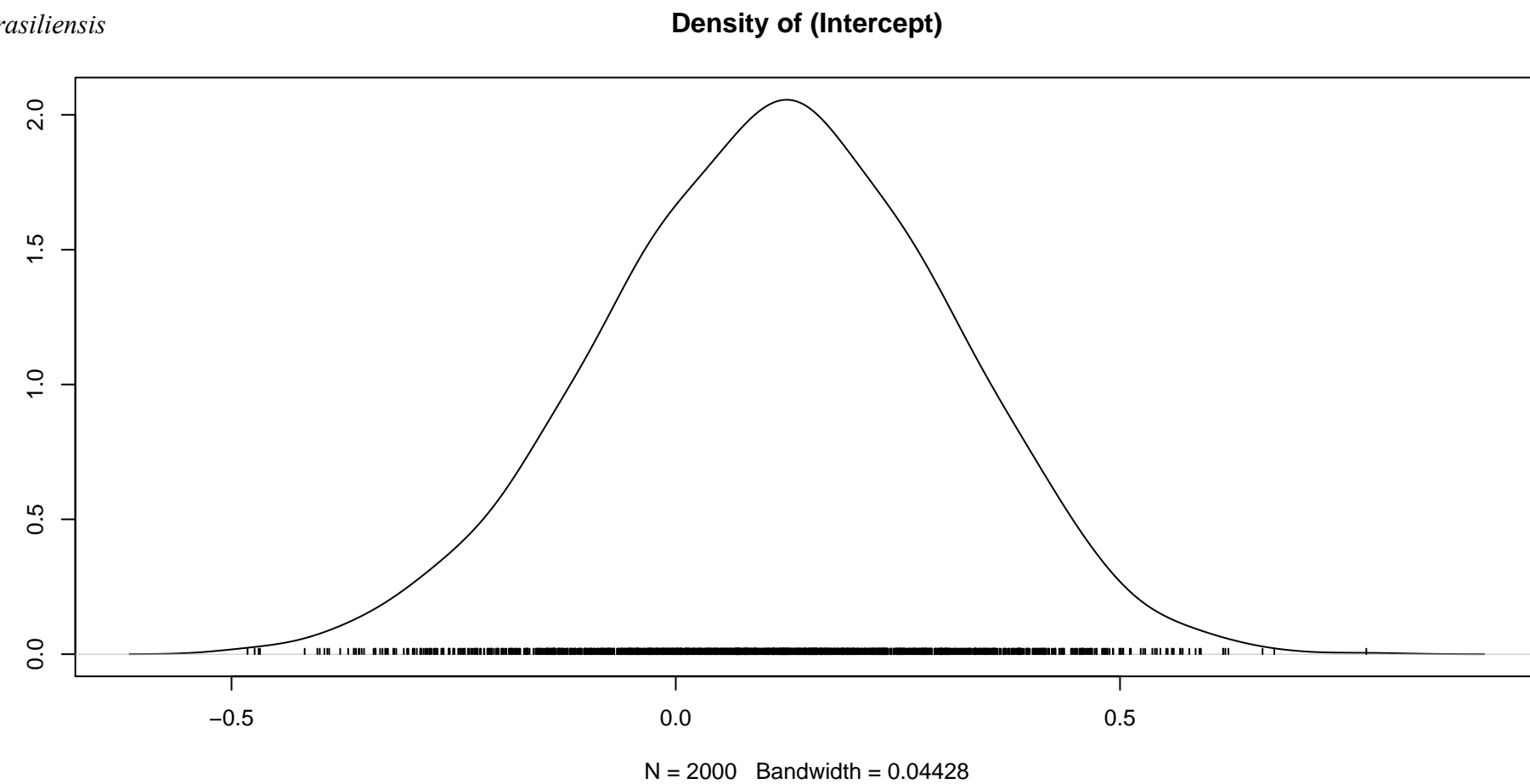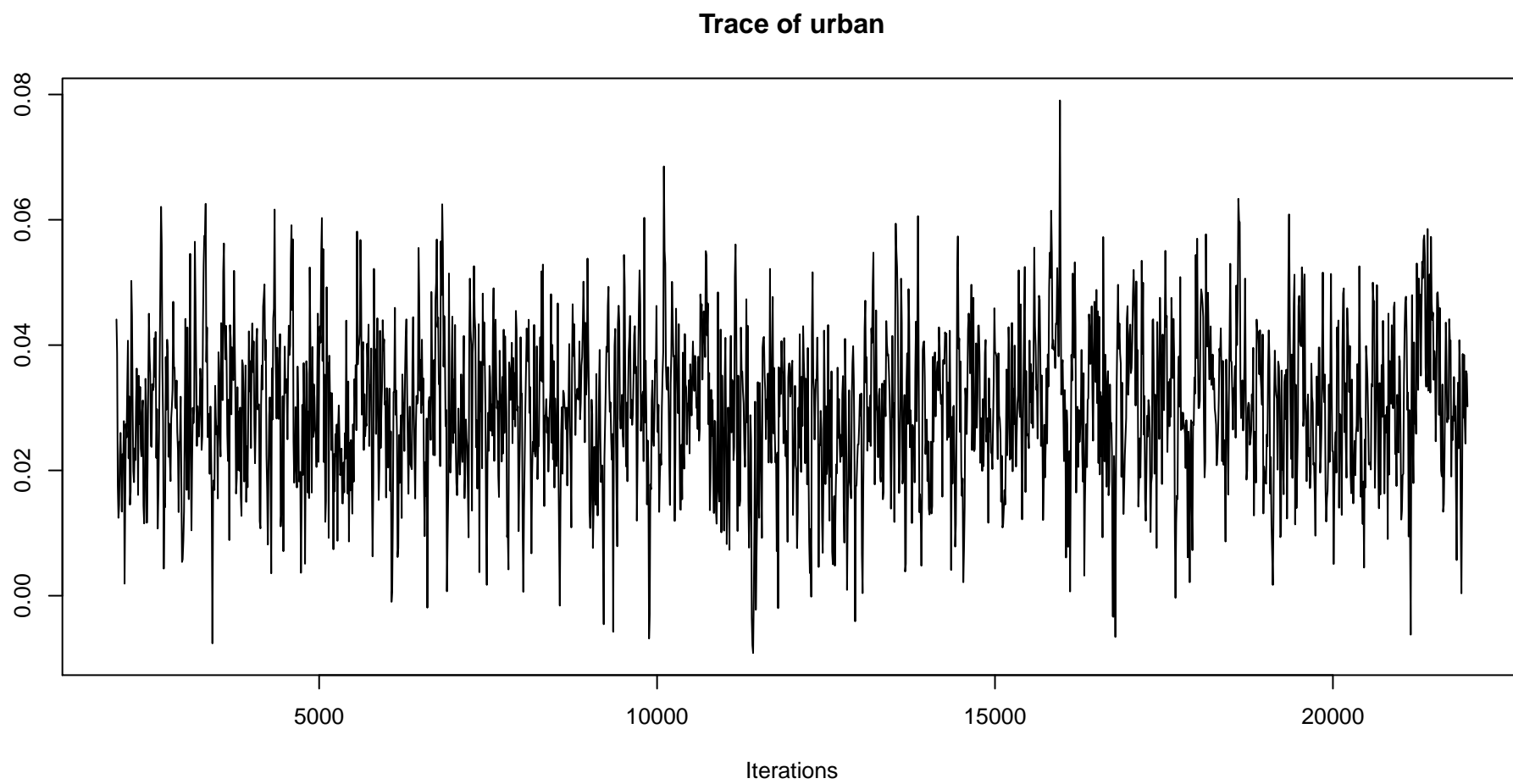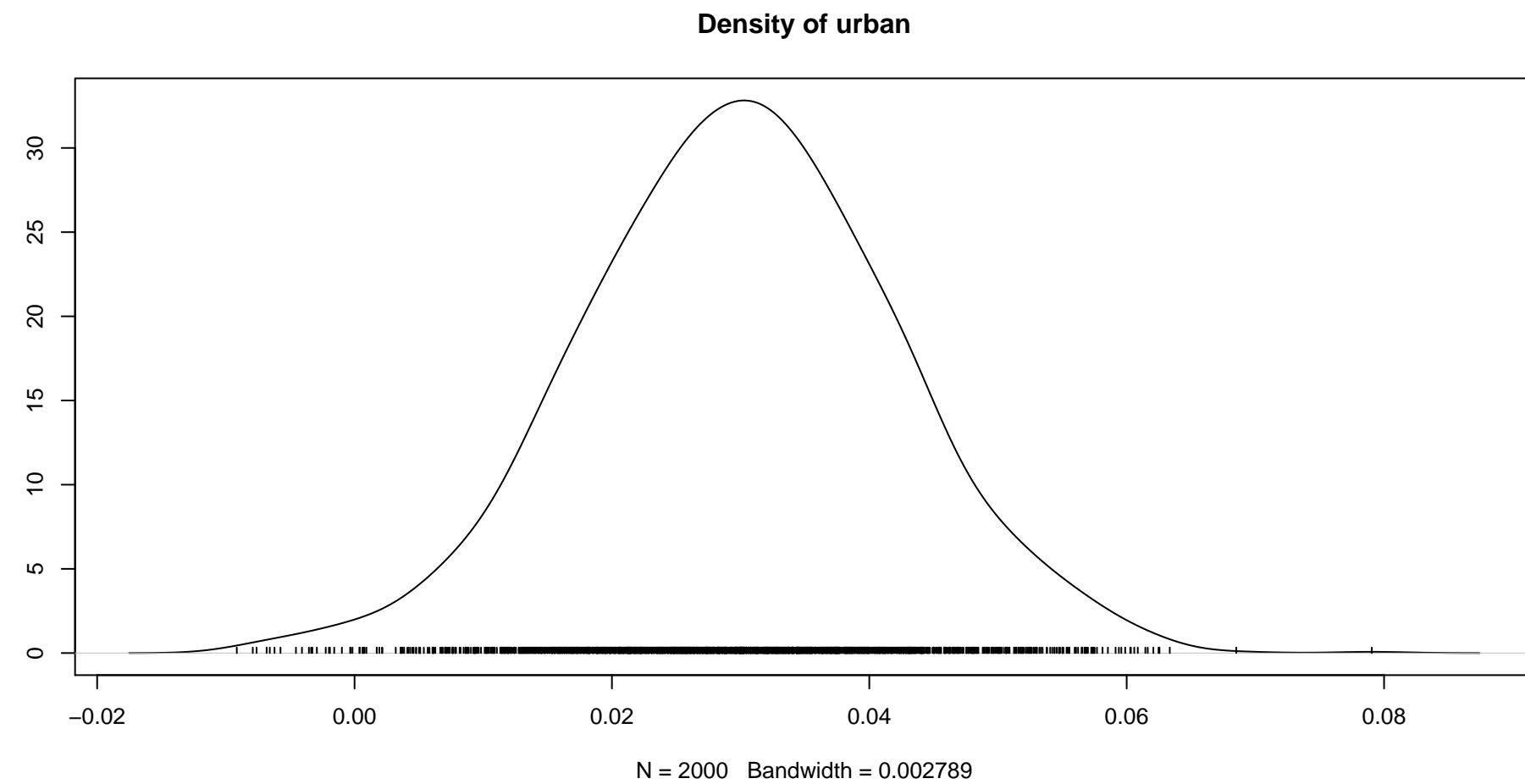

Supplement: Supplementary file 2 [file ECE3-8-667-s002.pdf]
