# Supplementary material for: Separating the effects of water quality and urbanization on temperate insectivorous bats at the landscape scale
Source: Ecol Evol. 2017 Dec 3;8(1):667–78. doi: 10.1002/ece3.3693 (PMC5756845; doi:10.1002/ece3.3693)
Supplement: Supplementary file 3 [file ECE3-8-667-s003.docx]

**Supporting Information**

Figure S1 Species-specific trace plots of generalized linear models using Markov Chain Monte Carlo simulation modeling bat activity against water quality. Comparisons were made between “excellent” and other water quality categories (“good,” “good-fair,” “fair”). Other covariates included in the model were not shown. Each model had 20000 simulations with a thinning interval of 10. Simulated generalized linear model’s regression estimates were plotted against each simulation’s iteration on the left for each independent variable. The density of regression estimates for each independent variable after thinning was plotted on the right.

Figure S2 Species-specific trace plots of generalized linear models using Markov Chain Monte Carlo simulation modeling bat activity against urban land cover. Other covariates included in the model were not shown. Each model had 20000 simulations with a thinning interval of 10. Simulated generalized linear model’s regression estimates were plotted against each simulation’s iteration on the left for each independent variable. The density of regression estimates for each independent variable after thinning was plotted on the right.
